# Real‐Time Visualisation of Reaction Kinetics and Dynamics: Single‐Molecule Insights into the Iminium‐Catalysed Diels–Alder Reaction

**DOI:** 10.1002/anie.202506535

**Published:** 2025-09-09

**Authors:** Minsoo Park, Yongdeok Ahn, Juhyeong Cho, Juhee Jang, Wonhee J. Lee, Sangwon Seo, Sunggi Lee, Daeha Seo

**Affiliations:** ^1^ Department of Physics and Chemistry DGIST Daegu 42988 Republic of Korea; ^2^ Department of Chemistry Pohang University of Science and Technology (POSTECH) Pohang 37673 Republic of Korea

**Keywords:** Diels–Alder reaction, Hidden Markov Model, Real‐time, Secondary amine catalyst, Single‐molecule imaging

## Abstract

Investigation of the fundamental microscopic processes occurring in organic reactions is essential for optimising both organocatalysts and synthetic strategies. In this study, single‐molecule fluorescence microscopy was employed to study the Diels–Alder reaction catalysed by a first‐generation MacMillan catalyst, providing direct insights into its kinetic dynamics. This reaction proceeds via a series of reversible processes under equilibrium conditions (**S** ⇄ **IM_1_** ⇄ **IM_2_** → **P**, IM_1_ and IM_2_: N,O‐acetal and iminium ion intermediates, respectively). The individual reaction trajectories of single molecules were directly observed in real‐time, and the kinetic transitions between the different states were quantitatively analysed using a hidden Markov model, thereby enabling precise determination of the kinetic rate constants and transition probabilities at the single‐molecule level. In particular, the unique structural features of the MacMillan catalyst were probed to reveal how specific interactions stabilise the reaction intermediates and influence their kinetic behaviours. These findings highlight the importance of single‐molecule fluorescence microscopy in understanding the fundamental mechanisms of organic reactions and guiding the rational design of more effective catalysts.

## Graphical abstract

Optical microscopy is commonly used to observe simple turn‐on/off phenomena in chemical reactions. A method is presented for the real‐time monitoring of diverse reaction pathways at the single‐molecule level using optical microscopy. This technique enables detailed kinetic and dynamic studies to be performed, providing new insights into catalyst design and the elucidation of reaction mechanisms.

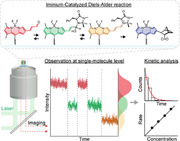

## Introduction

Understanding the kinetics of organic reactions and elucidating their mechanisms are central goals in organic chemistry, as they enable the rational design of novel reactions and catalysts.^[^ ^1^, ^2^, ^3^ ^]^ Traditionally, these mechanisms have been studied by investigating multiple experimental variables, and have subsequently been refined. However, the conventional ensemble averaging approach, which involves averaging the outcomes over time and across many molecules, often obscures dynamic processes. While recent single‐molecule methods based on surface‐bound molecular probes or electrode‐positioned molecules have provided valuable mechanistic insights, their application can be limited by technical constraints, such as the requirement for conductive substrates, electrochemical compatibility, vacuum conditions, or specialised interfaces, potentially restricting their adaptability to certain solvents and reaction environments.^[^ ^4^, ^5^, ^6^, ^7^, ^8^, ^9^ ^]^ In contrast, fluorescence‐based single‐molecule microscopy enables the real‐time observation of freely diffusing molecules in solution without requiring specialised surfaces or vacuum conditions. However, its successful implementation critically depends on both the solvent purity and the optical transparency. It is therefore apparent that each approach possesses unique advantages and limitations from the viewpoints of their solvent and environmental compatibilities.

Recent advancements in optical microscopy, which can realise a millisecond (ms) temporal resolution and a nanometre (nm) spatial resolution, have enabled the real‐time observation of single‐molecule biochemical processes.^[^ ^10^, ^11^, ^12^, ^13^ ^]^ Although single‐molecule studies have significantly advanced our understanding of enzymatic mechanisms, their application to conventional organic reactions remains relatively underexplored. The abovementioned recent developments in optical microscopy, therefore, show considerable potential for use in the study of organic reactions that occur on timescales ranging from ms to s, which could consequently enable the direct observation of transient structural rearrangements and subtle dynamic events.^[^ ^14^, ^15^, ^16^, ^17^, ^18^, ^19^ ^]^ Since spatiotemporal resolution is subject to a trade‐off, careful experimental design is essential to effectively optimise these parameters.

**Figure 1:**
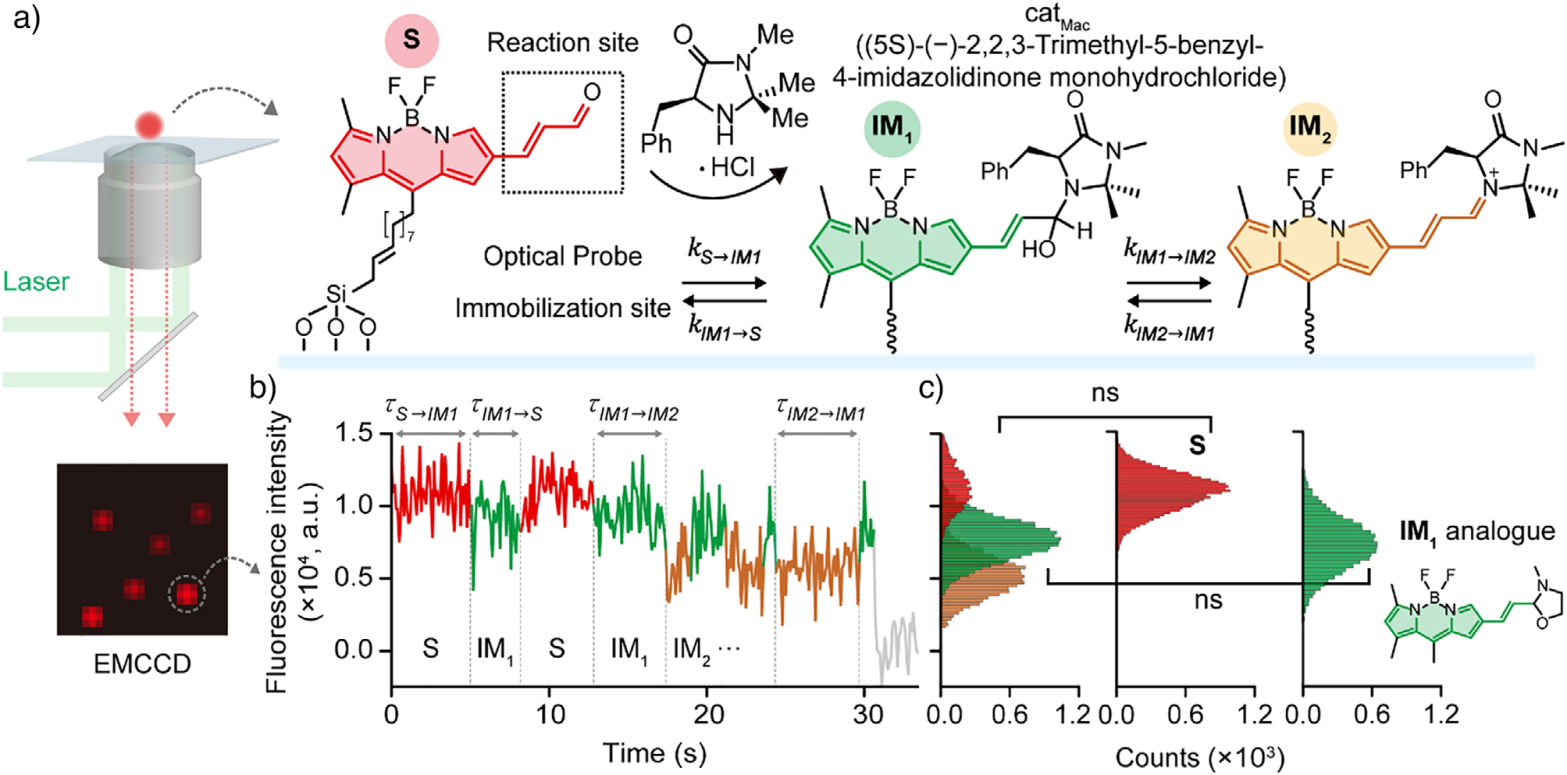
Experimental design for observing individual molecules during iminium ion formation in the presence of a MacMillan catalyst. a) Probe design for observation of the chemical reaction at the single‐molecule level, which incorporates the optical probe (BODIPY), an α,β‐enal moiety for the Diels–Alder reaction catalysed by cat_Mac_, and ‐Si(OEt)_3_ groups for immobilisation. The functionalised optical probe was immobilised on a glass slide and monitored using TIRF microscopy during the chemical reaction with cat_Mac_. b) Variation in the fluorescence intensity over time, allowing the formation of iminium ions to be monitored. Time traces for the individual probes were observed and separated by HMM analysis to provide details regarding the molecular kinetics via quantum chemical calculations. c) Intensity histograms showing the distribution of states over the time trajectories during the reaction performed in the presence of cat_Mac_. The fluorescence intensities of S and the IM_1_ analogue were compared with the states inferred by HMM analysis. The paired fluorescence intensities showed no statistically significant variations, specifically between S and its HMM‐estimated S, as well as between the IM_1_ analogue and its HMM‐estimated IM_1_. ns, not significant.

In this study, single‐molecule fluorescence microscopy is employed to investigate the elementary steps of an organic reaction using a fluorescent substrate as an optical probe (Figure 1a). By observing the real‐time reaction intermediates, kinetics, and structural dynamics, this approach is expected to offer a novel methodology for studying organic reaction mechanisms at the single‐molecule level. Moreover, the insights gained could facilitate the design of more efficient catalysts and reaction strategies.

## Results and Discussion

To develop a single‐molecule fluorescence microscopy approach for investigating the elementary steps of an organic reaction, a well‐known system was selected. This system incorporates the first‐generation MacMillan catalyst, (5*S*)‐(−)‐2,2,3‐trimethyl‐5‐benzyl‐4‐imidazolidinone monohydrochloride (referred to hereafter as cat_Mac_), and an α,β‐unsaturated aldehyde substrate bearing a BODIPY (4,4‐difluoro‐4‐bora‐3a,4a‐diaza‐s‐indacene) fluorophore. ^[^ ^20^, ^21^, ^22^ ^]^

This particular reaction system was chosen based on several experimental and mechanistic considerations. Firstly, the reaction proceeds at a moderate rate with minimal side reactions, typically reaching completion within a few hours under batch conditions (Figure S1). This slow reaction allows temporal resolution of all key intermediate steps under standard fluorescence imaging conditions.

Secondly, the reaction mechanism is well‐documented and involves changes in the conjugation length and electronic state of the dye as the reaction proceeds, leading to detectable optical variations.^[^ ^23^ ^]^ Previous kinetic studies have identified two plausible intermediates: i) A hemiaminal species (corresponding to the intermediate later referred to as **IM_1_**), which is formed via the nucleophilic attack of cat_Mac_ on the aldehyde; and ii) an iminium ion (later referred to as **IM_2_**), which is generated via an acid‐catalysed dehydration protocol.^[^ ^24^, ^25^, ^26^ ^]^ Although these intermediates are typically transient and difficult to isolate in ensemble experiments, their existence and kinetic relevance have been extensively supported by classical kinetic and mechanistic studies. Furthermore, stabilisation of these intermediates by intramolecular interactions between the fluorophore and the aromatic ring of cat_Mac_ has been reported, thereby highlighting their suitability for use in single‐molecule detection.

Building upon these established mechanistic foundations, the selected iminium‐catalysed system was expected to enable real‐time single‐molecule monitoring of intermediate formation, state transitions, and their kinetics, with the aim of providing detailed mechanistic insights at the molecular level.

More specifically, to ensure compatibility with single‐molecule fluorescence microscopy, the BODIPY‐α,β‐enal was designed to incorporate four key features. First, BODIPY was selected as the probe to ensure a high photostability and a reliable temporal resolution for fluorescence observations.^[^ ^27^, ^28^, ^29^, ^30^, ^31^ ^]^ Second, the α,β‐enal moiety was incorporated as the reactive group to facilitate changes in the fluorescence intensity via π–π conjugation alterations during the reaction. Third, a ‐Si(OEt)_3_ group was employed for immobilisation, enabling stable attachment to a glass surface to maximise the observation time and temporal resolution.^[^ ^19^, ^23^, ^32^, ^33^ ^]^ Fourth, a ‐(CH_2_)_7_‐ linker was incorporated to minimise steric hindrance between the substrate and the glass surface. In this case, BODIPY‐α,β‐enal was immobilised on a glass surface using a silane linker (<2 nm, e.g., ‐(CH_2_)_7_‐C_2_H_2_‐Si(OEt)_3_) to maintain stable fluorescence signals within the evanescent field during the total internal reflection fluorescence (TIRF) microscopy observations (Figure 1a). This probe was synthesised using the Vilsmeier–Haack reaction, wherein the silane linker preserves the optical properties of the fluorophore by not interfering with the π system (Figure S1).^[^ ^34^, ^35^, ^36^, ^37^, ^38^ ^]^ The reaction between the BODIPY‐α,β‐enal substrate (**S**) and cat_Mac_ proceeds as a multi‐step equilibrium process involving two intermediates, namely the N,O‐acetal **IM_1_** and the iminium ion **IM_2_**. Upon the addition of cyclopentadiene, the final product (**P**) is formed irreversibly (**S** ⇄ **IM_1_** ⇄ **IM_2_** → **P**). In this system, changes in the fluorescence signals occur owing to partial distortion of the π–π conjugation, which alters the conjugation length and shifts the fluorescence intensity.^[^ ^17^, ^39^, ^40^ ^]^

At the ensemble level, the designed Diels–Alder reaction was found to proceed at a sufficiently low rate under ambient conditions in the presence of the catalyst and the diene (Figure S1).^[^ ^41^ ^]^ This reaction induced detectable optical changes, and the maximum absorption wavelengths (*λ*_max_) of species **S** and **P** were determined to be 535 and 508 nm, respectively. Interestingly, despite the confirmed presence of multiple intermediates (i.e., **IM_1_** and **IM_2_**), a clear isosbestic point was observed in the ensemble fluorescence spectra. This indicates that these intermediates are likely too short‐lived or sparsely populated to significantly perturb the overall spectral balance in the bulk measurements. It was, therefore, anticipated that the intermediates could be effectively monitored and directly observed at the single‐molecule level, underscoring the unique advantage of single‐molecule fluorescence microscopy for revealing transient states, interconversion pathways, and detailed reaction kinetics that remain invisible to conventional ensemble approaches.

**Figure 2:**
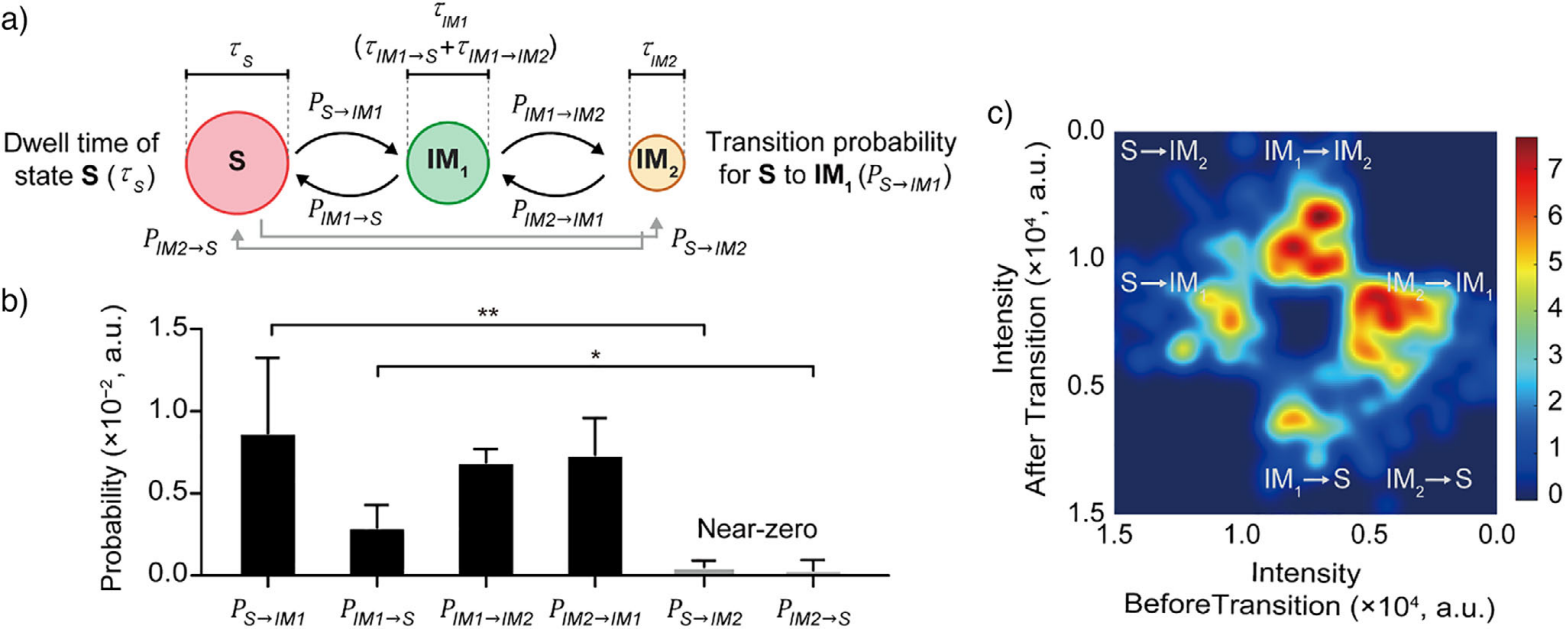
Transition probabilities for the different states present during the chemical reaction performed in the presence of cat_Mac_, as defined using HMM. a) Schematic illustration of the hidden Markov process. The molecule exhibits three different states, namely **S**, **IM_1_**, and **IM_2_**. In the three‐state system, the transition probability (*P*) indicates the probability of a molecule transitioning from its current state to the next state. b) Six transition probabilities for the three states changing to any other state ($P_{S\to IM1},\;P_{S\to IM2},\;P_{IM1\to S},P_{IM2\to S}\;P_{IM1\to IM2}\;P_{IM2\to IM1}$). The probabilities of transitions occurring from **S** to **IM_2_** and from **IM_2_** to **S** are practically negligible, indicating that four types of transitions exist, namely **S** ⇄ **IM_1_** and **IM_1_** ⇄ **IM_2_**. c) The corresponding transition density plot analysis for [cat_Mac_] = 15.0 mM was derived from the three‐state data sets, where the *x* and *y* coordinates correspond to the intensity before and after each transition, respectively. The scale bar indicates the relative densities of the state‐to‐state transitions. The gradient from blue to red corresponds to an increasing transition density, where blue denotes regions of low‐density transitions, and red emphasises regions of high‐density transitions. **p<0.01; *p<0.05.

**Figure 3:**
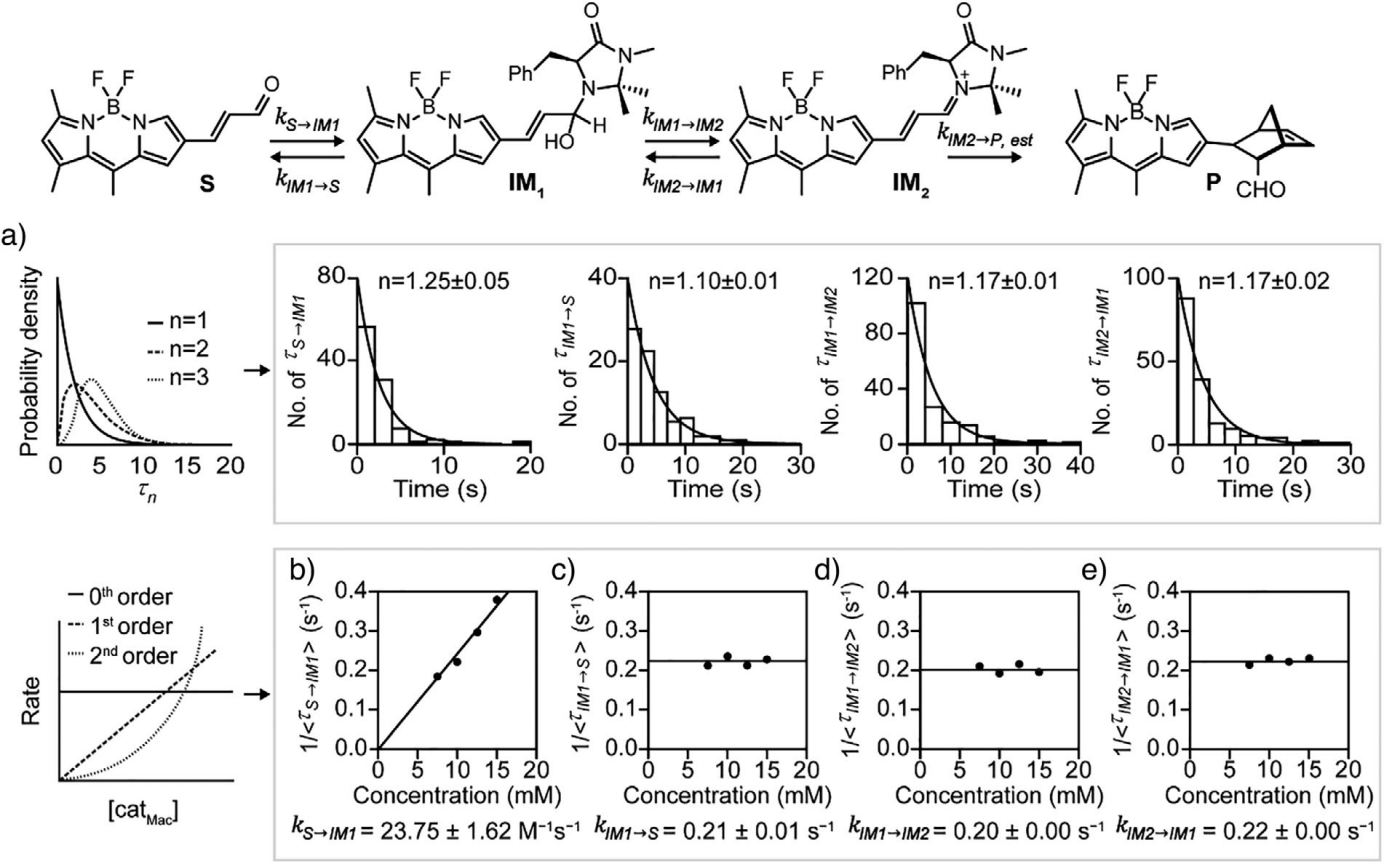
Dwell time distribution and reaction kinetics at the single‐molecule level. All experiments were performed in an ethanol solution with [cat_Mac_] = 15.0 mM. a) The distributions of the dwell time in each elementary step are fit with an exponential decay, t^n−1^exp(−kt). The *n* values are close to 1, as derived from the exponential fit, indicating that the reaction progresses via a sequential equilibrium pathway and all transitions follow a single kinetic step. b) Plots of the reciprocals of the mean dwell times for the formation of IM_1_ exhibit a linear dependence on the catalyst concentration. The reciprocal of the mean dwell time ($\tau_{S\to IM1}$) for the first step was used to calculate the rate constant ($k_{S\to IM1}$) for IM_1_ formation, which was determined to be 23.75 ± 1.62 M^−1^ s^−1^, using 1/(<$\tau_{S\to IM1}$>) = $k_{S\to IM1}$[cat_Mac_]. c), d), e) The reciprocals of the mean dwell times <$\tau_{IM1\to S}$>^−1^, <$\tau_{IM1\to IM2}$>^−1^, and <$\tau_{IM2\to IM1}$>^−1^ are independent of [cat_Mac_]. Averaged $k_{IM1\to S}$ = 0.21 ± 0.01 s^−1^, averaged $k_{IM1\to IM2}$ = 0.20 ± 0.00 s^−1^, and averaged $k_{IM2\to IM1}$ = 0.22 ± 0.00 s^−1^. Each data point represents an average calculated from ∼200 trajectories.

Thus, for single‐molecule imaging, the BODIPY substrate, **S**, was immobilised on a glass surface using silane chemistry, as described above, and subsequently observed in ethanol as the solvent. Notably, ethanol was selected owing to its ability to form hydrogen bonds with BODIPY, thereby ensuring a high photostability for this substrate and a relatively stable fluorescence signal.^[^ ^28^ ^]^ This setup enabled a sub‐second temporal resolution (∼100 ms) and long observation times (≤1 min) without photo‐blinking (Figure S2). The reaction was monitored using TIRF microscopy with 488 nm laser excitation, and the intensity trajectories (I(*t*)) were recorded over 1 min. Bleached signals were excluded from the analysis. To analyse the **IM** states, the hidden Markov model (HMM) algorithm was applied to the I(*t)* trajectories. HMM analysis revealed key aspects of the reaction, including its reversibility, the presence of intermediates, intermediate dwell times (τ*_s_*), and the transition probabilities ($P_{n\to m}$, *n* and *m* = **S**, **IM_1_**, and **IM_2_**) and rate constant (*k*) between the different states (see Figures 1, 2, and 3).^[^ ^42^, ^43^, ^44^, ^45^ ^]^

Following treatment with cat_Mac_, the single‐molecule trajectories revealed three statistically distinguishable signal states (i.e., **S**, **IM_1_**, and **IM_2_**; Figure S3) that exhibited reversible transitions indicative of a multi‐step equilibrium process (see Figures 1b, S4, and Movie S1). To validate the HMM‐inferred states, the fluorescence intensities of two species (i.e., **S** and the synthetic analogue of **IM_1_**) were compared with their respective HMM‐estimated counterparts (due to instability, an analogue for **IM_2_** could not be synthesised). The results showed no statistically significant differences between the paired fluorescence intensities (**S** versus HMM‐estimated **S**; **IM_1_** analogue versus HMM‐estimated **IM_1_**; Figure 1c), thereby supporting the existence of **S** and **IM_1_** (N,O‐acetal), along with a reversible equilibrium between these species. Although the exact identity of **IM_2_** could not be confirmed experimentally, its presence was inferred based on its reversible interconversion with **IM_1_** and its strong consistency with established mechanistic studies, which propose the iminium ion as the likely intermediate. The number of states was subsequently determined using the Bayesian Information Criterion, based on>200 trajectories (Figure S5).^[^ ^45^, ^46^, ^47^ ^]^ However, owing to the sub‐second temporal resolution (∼100 ms) of this single‐molecule imaging approach, rapid events such as protonation/deprotonation and conformational changes (on the ns–µs scale) could not be resolved. Consequently, analysis was limited to relatively long‐lived states that were detectable within this timescale.^[^ ^48^, ^49^ ^]^ Notably, this temporal resolution constraint represents a fundamental trade‐off with rigorous chemical identification methods such as single‐molecule spectroscopy, rendering it impractical to conclusively identify reaction intermediates that exist on sub‐second timescales (for a detailed discussion, see Note S1).

Subsequently, the state‐to‐state transition probabilities ($P_{n\to m}$) for the three‐state system (**S**, **IM_1_**, and **IM_2_**) were determined, wherein the transition probability $P_{S\to IM1}$ represents the likelihood that a molecule currently in state **S** will transition to state **IM_1_** in the subsequent time step (Figure 2a). Among over 800 transitions that were analysed herein, four main types of transitions were observed, namely **S** ⇄ **IM_1_** and **IM_1_** ⇄ **IM_2_**, while direct transitions from **S** to **IM_2_** (and vice versa) were extremely rare. For instance, $P_{S\to IM1},$ $P_{IM1\to S}$, and $P_{S\to IM2}$ were determined to be 8.8×10^−3^, 2.5×10^−3^, and 1.7×10^−4^, respectively (Figures 2b,c and S6). These values confirm that the observed transitions predominantly involved only two sequential pathways, i.e., **S** ⇄ **IM_1_** and **IM_1_** ⇄ **IM_2_**, while direct transitions such as **S** ⇄ **IM_2_** were not significantly detected.^[^ ^50^, ^51^ ^]^

To further assess whether these transitions (**S** ⇄ **IM_1_** and **IM_1_** ⇄ **IM_2_**) represent single‐step processes or involve hidden intermediates, the dwell time (*τ*) distributions were analysed for the various states. These dwell‐time distributions closely matched the single‐exponential fits, indicating that, within the temporal resolution evaluated herein (∼100 ms), the observed transitions occur as apparent single‐step processes without kinetically resolvable sub‐steps. This behaviour is consistent with a Poisson process, suggesting no hidden intermediates under the current experimental conditions (Figures 3a and S7). These results, together with advanced statistical modelling, indicate that the reaction follows a sequential equilibrium process (**S** ⇄ **IM_1_** ⇄ **IM_2_**), and that all transitions are governed by a single kinetic step.^[^ ^52^, ^53^ ^]^

A key advantage of these results is the ability to obtain detailed temporal and kinetic information for each elementary step (Figures 1, 2, and 3b). More specifically, in the initial step, the nucleophilic 2°‐amine catalyst attacks the immobilised α,β‐enal **S** to afford hemi‐aminal **IM_1_**, wherein the rate is proportional to the catalyst concentration ([cat_Mac_]). Consequently, the reciprocal of the mean dwell time of **S** (<τ_*S*_>) shows a linear increase with an increase in [cat_Mac_], yielding a rate constant ($k_{S\to IM1}$) of 23.75 ± 1.62 M^−1^ s^−1^ based on the first‐order kinetic law, 1/(<$\tau_{S\to IM1}$>) = $k_{S\to IM1}$[cat_Mac_] (Figure 3b).^[^ ^52^, ^53^, ^54^ ^]^ In contrast, the rates of the other equilibrium reactions were unaffected by changes in [cat_Mac_], consistent with zero‐order kinetics at [H^+^] > 7.5 mM (Figures 3c,d,e and S8). Notably, the elimination of water and the 2°‐amine catalyst from hemi‐aminal **IM_1_** represents a unimolecular process. Although hydrolysis of the iminium ion (**IM_2_**) depends on the water concentration, the use of ethanol as a solvent results in a constant water concentration. In addition, the kinetic data align well with the well‐established mechanism of iminium ion (**IM_2_**) formation between aldehydes and 2° amines.

**Figure 4:**
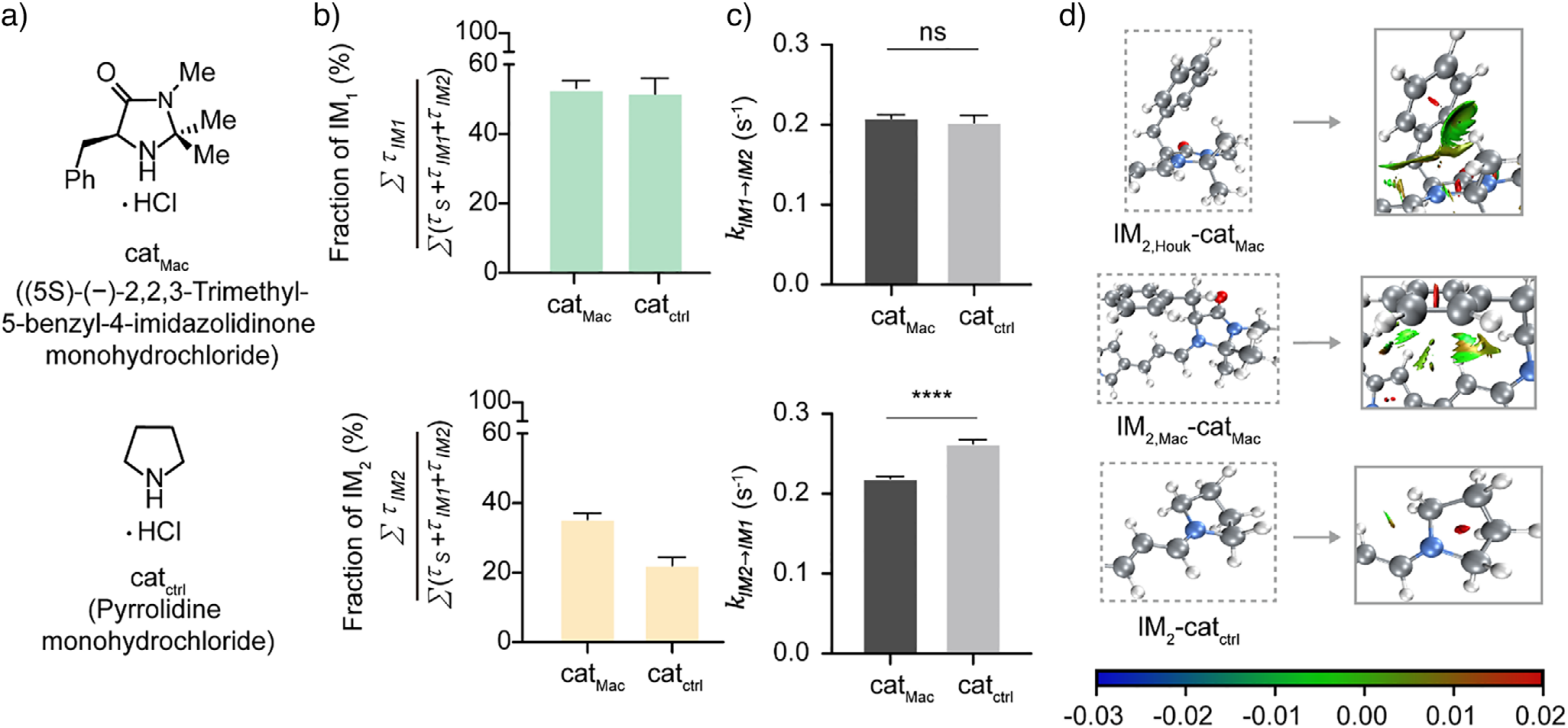
Statistical analysis of the relative proportions of each state during iminium ion (**IM_2_**) formation, and NCI analyses of the iminium ion intermediates (**IM_2_**). a) Proportions of the total dwell times of **IM_1_** and **IM_2_** within the observed reaction trajectories in the presence of 7.5 mM cat_Mac_ and cat_ctrl_. The addition of cat_Mac_ resulted in a 1.59‐fold increase in the proportion of **IM_2_**, while the fraction of **IM_1_** remained unchanged. b) Rate constants of the elementary steps occurring in the presence of cat_Mac_ and cat_ctrl_. Using cat_Mac_, a lower rate constant was observed for the transition from **IM_2_** to **IM_1_**. c) Computed structures and NCI analysis of the core scaffolds of several iminium intermediates. d) The Houk conformer, the MacMillan conformer, and the iminium ion generated using cat_ctrl_. IM_2,Houk_‐cat_Mac_, and IM_2,Mac_‐cat_Mac_ are stabilised by CH–π and cation–π interactions. Calculations were performed on a simplified model in which the long alkyl chains were excluded. The scale bar represents the strength of the interactions, with negative values (blue) indicating attractive forces, near‐zero values (green) denoting weak van der Waals interactions, and positive values (red) representing repulsive forces. ****p<0.0001; ns, not significant.

In the reaction performed using cat_Mac_, the iminium intermediate (**IM_2_**) is crucial as it activates the substrate by lowering the lowest unoccupied molecular orbital (LUMO). This increases the electrophilicity and thereby enhances the reaction rate, while also serving as a tool to control the enantioselectivity (Figure S9).^[^ ^55^, ^56^ ^]^ To empirically and quantitatively assess these effects, an identical set of single‐molecule experiments were conducted using a control 2°‐amine catalyst, namely pyrrolidine monohydrochloride (cat_ctrl_), which lacks key functional groups. More specifically, this catalyst lacks the aromatic (C_6_H_5_‐) and methyl (CH_3_‐) substituents that are responsible for stabilising the intramolecular interactions (e.g., CH–π and cation–π interactions) and spatial orientation in cat_Mac_ (Figure 4a). Owing to this simplified structure, cat_ctrl_ is not expected to promote significant non‐covalent interactions or steric effects, thereby allowing these features to contribute to the formation and stabilisation of **IM_2_**. Upon comparing the kinetic parameters obtained using cat_ctrl_ with those obtained using cat_Mac_, it was observed that cat_Mac_ increased the fraction of **IM_2_** by 1.59‐fold, while the fraction of **IM_1_** remained unchanged, and the rate constant $k_{IM2\to IM1}$ decreased (Figure 4b,c). This observation, supported by density functional theory (DFT) calculations and non‐covalent interaction (NCI) analysis, indicates that CH–π and cation–π interactions stabilise **IM_2_** via secondary interactions with the *syn*‐methyl group (IM_2,Houk_‐cat_Mac_) and the iminium moiety (IM_2,Mac_‐cat_Mac_) of the catalyst (cat_Mac_), respectively (Figure 4d). These interactions enable a more efficient reaction with the diene at the active site. Subsequently, the Diels–Alder reaction was investigated upon the addition of a diene, namely cyclopentadiene. Because of the limitations of this single‐molecule setup, the optical signal of **P** (*I_P_*) was statistically indistinguishable from that of **IM_1_** (*I*_*IM*1_), thereby complicating state differentiation (Figure S10). However, the rate constant for product formation ($k_{IM2\to P,\;est}$) was inferred from the experimental results described below.

**Figure 5:**
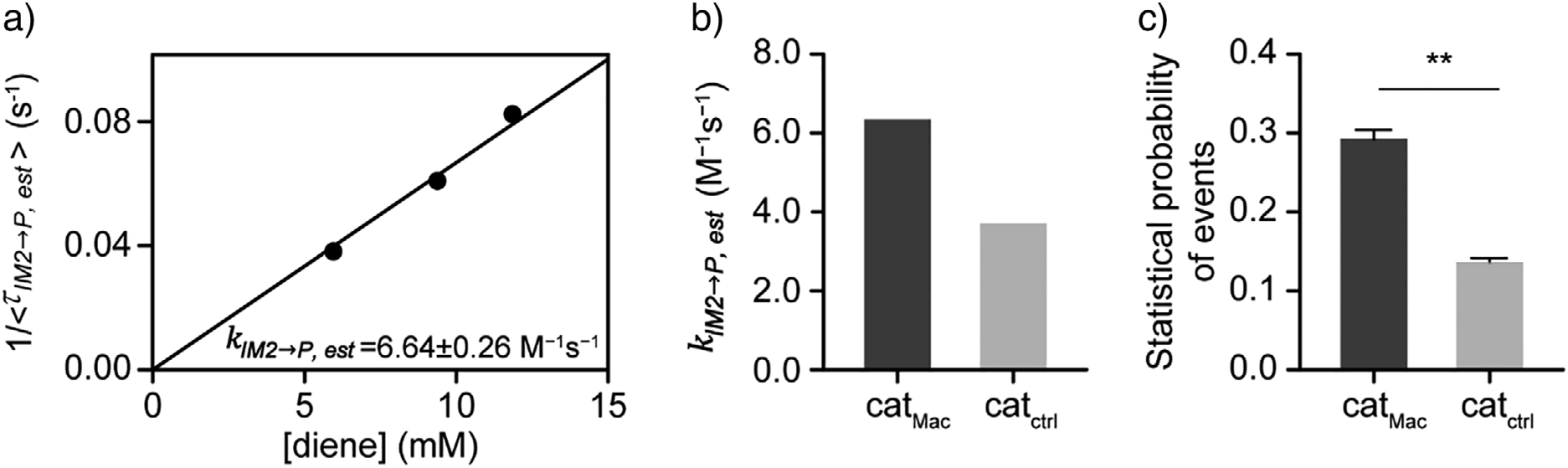
Kinetic studies of the organocatalyst‐induced Diels–Alder reaction. a) Plots representing the reciprocals of the mean dwell times ($\tau_{IM2\to P,\;est}$) for the Diels–Alder reaction conducted in the presence of cat_Mac_. The rate exhibits a linear dependence on the diene concentration. b) Calculated values of $k_{IM2\to P,\;est}$ = 6.64 ± 0.26 M^−1^ s^−1^ (in the presence of cat_Mac_) and 3.82 ± 0.24 M^−1^ s^−1^ (in the presence of cat_ctrl_). c) Frequency of event occurrence in the Diels–Alder reactions performed using cat_Mac_ and cat_ctrl_. For cat_Mac_, the frequency of the Diels–Alder reaction increased 2.07‐fold compared with that observed for cat_ctrl_. Stabilisation of the iminium intermediate through secondary interactions with cat_Mac_ enhances its reaction efficiency. **p<0.01.

The proportion of **IM_2_** decreased in the presence of both cat_Mac_ and the diene, while the proportion of **IM_1_** increased in the presence of cat_Mac_ alone, suggesting contributions from both **IM_1_** and **P** (τ_*P*_) (Figure S11). In addition, using the Markov property, which asserts that the current state depends only on the previous state, it was possible to derive $k_{IM2\to P,\;est}$. Subsequently, the values of <$\tau_{IM1\to S}$>^−1^ and <$\tau_{IM1\to IM2}$>^−1^ remained constant and were identical to those obtained in the experiment performed using cat_Mac_ alone, thereby allowing the calculation of $k_{IM2\to P,\;est}$ based on the relationship τ_*IM*2_ = 1/($k_{IM2\to IM1}+k_{IM2\to P,\;est}$) (Figure S12). Furthermore, a plot of (<$\tau_{IM2\to P,\;est}$>^−1^) as a function of the diene concentration showed a linear dependence, confirming first‐order kinetics (Figure 5a). Considering these analyses, $k_{IM2\to P,\;est}$ was estimated for cat_Mac_, yielding a value that was 1.75‐fold higher than that associated with cat_ctrl_ (Figure 5b).^[^ ^56^, ^57^ ^]^ Additionally, the frequency of the Diels–Alder reaction was found to be 2.07‐fold higher in the presence of cat_Mac_ (c.f., cat_ctrl_; see Figure 5c), thereby suggesting that secondary interactions involving cat_Mac_ significantly stabilise the iminium intermediate and the corresponding transition state to enhance its reactivity, ultimately leading to an improved reaction efficiency.

In the context of enantioselective catalysis, identifying the structure of **IM_2_** is important for understanding the key reaction pathways leading to **P**. As previously discussed, the **IM_2_** species formed in the presence of cat_Mac_ predominantly exists in two conformers, as proposed by MacMillan (**IM_2,Mac_**; Mac conformer) and Houk (**IM_2,Houk_**; Houk conformer).^[^ ^22^, ^58^, ^59^, ^60^, ^61^, ^62^, ^63^ ^]^ If conformational transitions occur sufficiently slowly and each conformer exhibits distinct, optically distinguishable photophysical signatures, the single‐molecule fluorescence microscopy approach presented in this study offers significant potential for directly observing conformational dynamics in real‐time. Indeed, the obtained experimental data provide indirect yet supportive evidence for such conformational distinctions, in alignment with prior experimental reports. However, a notable discrepancy remains between theory and observation. Particularly, theoretical calculations predict an extremely low rotational barrier (∼6.0 kcal mol^−1^), indicating conformational interconversions at the microsecond or even nanosecond timescales, which are substantially faster than the current temporal resolution. Thus, at present, this issue remains unresolved, as discussed in Note S2. Addressing this challenge will not only deepen our mechanistic understanding of diverse organic reactions, but will also significantly broaden the applicability of single‐molecule methodologies.

## Conclusion

This study demonstrates the ability of single‐molecule fluorescence microscopy to elucidate reaction mechanisms at an unprecedented spatiotemporal resolution. By employing the MacMillan catalyst, key intermediates (IM_1_ and IM_2_) were identified, and it was possible to directly observe that these reactions exist in dynamic equilibrium. In addition, hidden Markov model analysis enabled precise determination of the kinetic constants for each elementary step, while density functional theory calculations highlighted the roles of CH–π and cation–π interactions with the MacMillan catalyst in stabilising the iminium intermediate and enhancing its reactivity, compared with the case of the control catalyst. Beyond mechanistic insights, this study underscores the broader applicability of kinetic methodologies in mechanistic investigations. Unlike previous fluorescence‐based studies, which primarily tracked turn‐on/turn‐off signals or single‐step transformations, this approach enables the real‐time observation of multi‐step equilibria and quantitative kinetic profiling at the single‐molecule level. The ability to resolve and analyse multiple reaction intermediates, along with their state‐specific dynamics, offers a generalisable framework for dissecting complex organic reaction mechanisms with high precision. Overall, this work establishes a methodology that extends beyond the MacMillan‐catalysed reaction, providing a foundation for the real‐time kinetic visualisation of multi‐step organic transformations. These findings emphasise the importance of single‐molecule techniques in uncovering previously inaccessible mechanistic details and highlight the potential of kinetic profiling for guiding the rational design of catalysts and reaction conditions. Furthermore, this real‐time visualisation of organic reactions provides a methodological foundation for future analyses of their statistical and collective behaviours.

## Supporting Information

The authors have cited additional references within the Supporting information.^[^ ^64^, ^65^, ^66^, ^67^, ^68^, ^69^, ^70^, ^71^ ^]^ Supporting information includes the following contents: Experimental Section, Supporting Figures, Supporting Notes, NMR Spectra, Computational Details, Supporting Movie Legend, Figures S1–S12, Notes S1–S2, and Movie S1.

## Conflict of Interests

The authors declare no conflict of interest.

## Supporting information



Supporting Information

Supporting Information

## Data Availability

The data that support the findings of this study are available from the corresponding author upon reasonable request.

## References

[1] S. J. Liu , Z. H. Chen , J. Y. Chen , S. F. Ni , Y. C. Zhang , F. Shi , Angew. Chem. Int. Ed. 2022, 61, e202112226. 10.1002/anie.202112226 34846087

[2] J. M. Tian , Y. H. Yuan , Y. Q. Tu , F. M. Zhang , X. B. Zhang , S. H. Zhang , S. H. Wang , X. M. Zhang , Chem. Commun. 2015, 51, 9979–9982. 10.1039/c5cc02765a 25998622

[3] J. M. Lassaletta , Nat. Commun. 2020, 11, 3787. 32728081 10.1038/s41467-020-17600-y PMC7391751

[4] C. Yang , Y. Li , S. Zhou , Y. Guo , C. Jia , Z. Liu , K. N. Houk , Y. Dubi , X. Guo , Nat. Chem. 2023, 15, 972–979. 37188972 10.1038/s41557-023-01212-2

[5] W. Hu , M. Li , W. Xiong , S. Zhou , Q. Zou , J. T. Lü , H. Tian , X. Guo , J. Am. Chem. Soc. 2024, 146, 17765–17772. 38902874 10.1021/jacs.4c03071

[6] J. Guan , C. Jia , Y. Li , Z. Liu , J. Wang , Z. Yang , C. Gu , D. Su , K. N. Houk , D. Zhang , X. Guo , Sci. Adv. 2018, 4, eaar2177. 29487914 10.1126/sciadv.aar2177 PMC5817934

[7] A. Riss , A. P. Paz , S. Wickenburg , H.‐Z. Tsai , D. G. De Oteyza , A. J. Bradley , M. M. Ugeda , P. Gorman , H. S. Jung , M. F. Crommie , A. Rubio , F. R. Fischer , Nat. Chem. 2016, 8, 678–683. 27325094 10.1038/nchem.2506

[8] X. Huang , C. Tang , J. Li , L.‐C. Chen , J. Zheng , P. Zhang , J. Le , R. Li , X. Li , J. Liu , Y. Yang , J. Shi , Z. Chen , M. Bai , H.‐L. Zhang , H. Xia , J. Cheng , Z.‐Q. Tian , W. Hong , Sci. Adv. 2019, 5, 1–7. 10.1126/sciadv.aaw3072 PMC6588380 31245539

[9] K. Vasilev , B. Doppagne , T. Neuman , A. Rosławska , H. Bulou , A. Boeglin , F. Scheurer , G. Schull , Nat. Commun. 2022, 13, 677. 35115513 10.1038/s41467-022-28241-8 PMC8813982

[10] W. B. Asher , P. Geggier , M. D. Holsey , G. T. Gilmore , A. K. Pati , J. Meszaros , D. S. Terry , S. Mathiasen , M. J. Kaliszewski , M. D. McCauley , A. Govindaraju , Z. Zhou , K. G. Harikumar , K. Jaqaman , L. J. Miller , A. W. Smith , S. C. Blanchard , J. A. Javitch , Nat. Methods. 2021, 18, 397–405. 33686301 10.1038/s41592-021-01081-y PMC8232828

[11] J. A. Rivera , G. Mao , A. Sabantsev , M. Panfilov , Q. Hou , M. Lindell , C. Chanez , F. Ritort , M. Jinek , S. Deindl , Science 2024, 385, 892–898. 39172826 10.1126/science.adn5371

[12] S. Jin , Y. Ahn , J. Park , M. Park , S. C. Lee , W. J. Lee , D. Seo , Adv. Sci. 2024, 11, 2306849. 10.1002/advs.202306849 PMC11304332 38828676

[13] R. H. Goldsmith , W. E. Moerner , Nat. Chem. 2010, 2, 179–186. 20625479 10.1038/nchem.545 PMC2899709

[14] D. Yu , A. Garcia , S. A. Blum , K. D. Welsher , J. Am. Chem. Soc. 2022, 144, 14698–14705. 35867381 10.1021/jacs.2c04990

[15] Z. Su , J. S. Baskin , W. Zhou , J. M. Thomas , A. H. Zewail , J. Am. Chem. Soc. 2017, 139, 4916–4922. 28273420 10.1021/jacs.7b00906

[16] Y. Ahn , J. Park , M. Park , S. Jin , W. Jo , J. Kim , S. H. Cho , D. Seo , Chem 2022, 8, 2485–2497.

[17] C. Li , S. Duan , J. Yi , C. Wang , P. M. Radjenovic , Z. Tian , J. Li , Sci. Adv. 2020, 6, eaba6012. 32577524 10.1126/sciadv.aba6012 PMC7286666

[18] B. K. Yoo , O. H. Kwon , H. Liu , J. Tang , A. H. Zewail , Nat. Commun. 2015, 6, 8639. 26478194 10.1038/ncomms9639 PMC4667692

[19] N. M. Esfandiari , Y. Wang , J. Y. Bass , S. A. Blum , Inorg. Chem. 2011, 50, 9201–9203. 21863905 10.1021/ic2007952

[20] D. W. C. MacMillan , Nature 2008, 455, 304–308. 18800128 10.1038/nature07367

[21] R. Gordillo , J. Carter , K. N. Houk , Adv. Synth. Catal. 2004, 346, 1175–1185.

[22] M. C. Holland , S. Paul , W. B. Schweizer , K. Bergander , C. Mück‐Lichtenfeld , S. Lakhdar , H. Mayr , R. Gilmour , Angew. Chem. Int. Ed. 2013, 52, 7967–7971. 10.1002/anie.201301864 23794233

[23] A. Rybina , C. Lang , M. Wirtz , K. Grußmayer , A. Kurz , F. Maier , A. Schmitt , O. Trapp , G. Jung , D. P. Herten , Angew. Chem. Int. Ed. 2013, 52, 6322–6325. 10.1002/anie.201300100 23630097

[24] E. H. Cordes , W. P. Jencks , J. Am. Chem. Soc. 1963, 85, 2843–2848.

[25] K. Koehler , W. Sandstrom , E. H. Cordes , J. Am. Chem. Soc. 1964, 86, 2413–2419.

[26] Y. Ogata , A. Kawasaki , Tetrahedron 1964, 20, 1573–1578.

[27] S. C. Yeh , L. J. Wang , H. M. Yang , Y. H. Dai , C. W. Lin , C. T. Chen , R. J. Jeng , Chem.‐Eur. J. 2017, 23, 14747–14759. 28762579 10.1002/chem.201702285

[28] X. F. Zhang , G. Q. Zhang , J. Zhu , J. Fluoresc. 2019, 29, 407–416. 30675679 10.1007/s10895-019-02349-5

[29] S. Adhikari , J. Moscatelli , E. M. Smith , C. Banerjee , E. M. Puchner , Nat. Commun. 2019, 10, 3400. 31363088 10.1038/s41467-019-11384-6 PMC6667493

[30] E. Palao , G. Duran‐Sampedro , S. De La Moya , M. Madrid , C. García‐López , A. R. Agarrabeitia , B. Verbelen , W. Dehaen , N. Boens , M. J. Ortiz , J. Org. Chem. 2016, 81, 3700–3710. 27055068 10.1021/acs.joc.6b00350

[31] I. J. Arroyo , R. Hu , G. Merino , B. Z. Tang , E. Peña‐Cabrera , J. Org. Chem. 2009, 74, 5719–5722. 19572588 10.1021/jo901014w

[32] S. M. Canham , J. Y. Bass , O. Navarro , S. G. Lim , N. Das , S. A. Blum , Organometallics 2008, 27, 2172–2175.

[33] J. D. Ng , S. P. Upadhyay , A. N. Marquard , K. M. Lupo , D. A. Hinton , N. A. Padilla , D. M. Bates , R. H. Goldsmith , J. Am. Chem. Soc. 2016, 138, 3876–3883. 26944030 10.1021/jacs.6b00357

[34] A. Bessette , T. Auvray , D. Désilets , G. S. Hanan , Dalton Trans. 2016, 45, 7589–7604. 27045555 10.1039/c5dt04444h

[35] T. Ono , D. Koga , K. Yoza , Y. Hisaeda , Chem. Commun. 2017, 53, 12258–12261. 10.1039/c7cc07170a 29094113

[36] E. P. Utiel , L. M. Jiménez , I. Esnal , R. P. Montero , A. R. Agarrabeitia , I. García‐Moreno , J. Bañuelos , I. López‐Arbeloa , S. Moya , M. J. Ortiz , Dyes Pigm. 2017, 141, 286–298.

[37] A. Savoldelli , G. Magna , C. Di Natale , A. Catini , S. Nardis , F. R. Fronczek , K. M. Smith , R. Paolesse , Chem.‐Eur. J. 2017, 23, 14819–14826. 28833687 10.1002/chem.201702380

[38] A. K. Chatterjee , T. L. Choi , D. P. Sanders , R. H. Grubbs , J. Am. Chem. Soc. 2003, 125, 11360–11370. 16220959 10.1021/ja0214882

[39] P. Costa , D. Sandrin , J. C. Scaiano , Nat. Catal. 2020, 3, 427–437.

[40] H. Liu , W. Song , D. Gröninger , L. Zhang , Y. Lu , K. S. Chan , Z. Zhou , K. Rurack , Z. Shen , Commun. Biol. 2019, 2, 442. 31815197 10.1038/s42003-019-0682-1 PMC6883057

[41] K. A. Ahrendt , C. J. Borths , D. W. C. MacMillan , J. Am. Chem. Soc. 2000, 122, 4243–4244.

[42] N. R. Marzano , B. P. Paudel , A. M. van Oijen , H. Ecroyd , Sci. Adv. 2022, 8, eadd0922. 36516244 10.1126/sciadv.add0922 PMC9750156

[43] F. Persson , M. Lindén , C. Unoson , J. Elf , Nat. Methods 2013, 10, 265–269. 23396281 10.1038/nmeth.2367

[44] C. S. Smith , K. Jouravleva , M. Huisman , S. M. Jolly , P. D. Zamore , D. Grunwald , Nat. Commun. 2019, 10, 272. 30655518 10.1038/s41467-018-08045-5 PMC6336789

[45] D. S. White , S. Chowdhury , V. Idikuda , R. Zhang , S. T. Retterer , R. H. Goldsmith , B. Chanda , Nature 2021, 595, 606–610. 34194042 10.1038/s41586-021-03686-x PMC8513821

[46] Y. Takashima , Y. Komoto , T. Ohshiro , K. Nakatani , M. Taniguchi , J. Am. Chem. Soc. 2023, 145, 1310–1318. 36597667 10.1021/jacs.2c11260

[47] Y. Lee , J. Buchheim , B. Hellenkamp , D. Lynall , K. Yang , E. F. Young , B. Penkov , S. Sia , M. N. Stojanovic , K. L. Shepard , Nat. Nanotechnol. 2024, 19, 660–667. 38233588 10.1038/s41565-023-01591-0 PMC11229667

[48] C. Zhao , J. Diao , Z. Liu , J. Hao , S. He , S. Li , X. Li , G. Li , Q. Fu , C. Jia , X. Guo , Nat. Commun. 2024, 15, 8835. 39397019 10.1038/s41467-024-53179-4 PMC11471814

[49] J. Lee , B. T. Miller , A. Damjanović , B. R. Brooks , J. Chem. Theory Comput. 2014, 10, 2738–2750. 25061443 10.1021/ct500175m PMC4095908

[50] J. Y. Kim , C. Kim , N. K. Lee , Nat. Commun. 2015, 6, 6992. 25908552 10.1038/ncomms7992 PMC4421855

[51] S. A. McKinney , C. Joo , T. Ha , Biophys. J. 2006, 91, 1941–1951. 16766620 10.1529/biophysj.106.082487 PMC1544307

[52] Y. Zhang , P. Song , Q. Fu , M. Ruan , W. Xu , Nat. Commun. 2014, 5, 4238. 24963600 10.1038/ncomms5238

[53] M. P. Goldschen‐Ohm , D. S. White , V. A. Klenchin , B. Chanda , R. H. Goldsmith , Angew. Chem. Int. Ed. 2017, 129, 2439–2442. 10.1002/anie.201612050 PMC6166642 28116856

[54] Y. Qing , M. D. Liu , D. Hartmann , L. Zhou , W. J. Ramsay , H. Bayley , Angew. Chem. Int. Ed. 2020, 59, 15711–15716. 10.1002/anie.202005729 PMC7496719 32589803

[55] G. Evans , T. J. K. Gibbs , R. L. Jenkins , S. J. Coles , M. B. Hursthouse , J. A. Platts , N. C. O. Tomkinson , Angew. Chem. Int. Ed. 2008, 120, 2862–2865. 10.1002/anie.200705539 18311740

[56] S. Lakhdar , T. Tokuyasu , H. Mayr , Angew. Chem. Int. Ed. 2008, 47, 8723–8726. 10.1002/anie.200802889 18798186

[57] A. M. Costa , V. Cascales , A. Castro‐Alvarez , J. Vilarrasa , ACS Omega 2022, 7, 18247–18258. 35694469 10.1021/acsomega.1c07020 PMC9178767

[58] T. P. Golub , C. Merten , Phys. Chem. Chem. Phys. 2021, 23, 25162–25169. 34730148 10.1039/d1cp04497d

[59] M. C. Holland , J. B. Metternich , C. Mück‐Lichtenfeld , R. Gilmour , Chem. Commun. 2015, 51, 5322–5325. 10.1039/c4cc08520e 25434331

[60] J. C. Burley , R. Gilmour , T. J. Prior , G. M. Day , Acta Crystallogr. 2008, 64, C29–C29. 10.1107/S0108270107051396 18216449

[61] M. C. Holland , G. Berden , J. Oomens , A. J. H. M. Meijer , M. Schäfer , R. Gilmour , Eur. J. Org. Chem. 2014, 2014, 5675–5680.

[62] C. Sparr , R. Gilmour , Angew. Chem. Int. Ed. 2010, 49, 6520–6523. 10.1002/anie.201003734 20677308

[63] M. C. Holland , R. Gilmour , Angew. Chem. Int. Ed. 2015, 127, 3934–3943.

[64] R. G. Parr , Y. Weitao , in Density Functional Theory of Atoms and Molecules, Oxford University Press, New York, 1989.

[65] M. J. Frisch , G. W. Trucks , H. B. Schlegel , G. E. Scuseria , M. A. Robb , J. R. Cheeseman , G. Scalmani , V. Barone , G. A. Petersson , H. Nakatsuji , X. Li , M. Caricato , A. V. Marenich , J. Bloino , B. G. Janesko , R. Gomperts , B. Mennucci , H. P. Hratchian , J. V. Ortiz , A. F. Izmaylov , J. L. Sonnenberg , D. Williams‐Young , F. Ding , F. Lipparini , F. Egidi , J. Goings , B. Peng , A. Petrone , T. Henderson , D. Ranasinghe , et al., Gaussian 16, Revision C. 01, Gaussian, Inc., Wallingford CT, 2016.

[66] C. Lee , W. Yang , R. G. Parr , Phys. Rev. B. 1988, 37, 785–789. 10.1103/physrevb.37.785 9944570

[67] A. D. Becke , J. Chem. Phys. 1993, 98, 5648–5652.

[68] S. Grimme , J. Antony , S. Ehrlich , H. Krieg , J. Chem. Phys. 2010, 132, 154104. 20423165 10.1063/1.3382344

[69] F. Weigend , R. Ahlrichs , Phys. Chem. Chem. Phys. 2005, 7, 3297. 16240044 10.1039/b508541a

[70] A. V. Marenich , C. J. Cramer , D. G. Truhlar , Phys. Chem. B. 2009, 113, 6378–6396. 10.1021/jp810292n 19366259

[71] T. Lu , F. Chen , Comput. Chem. 2012, 33, 580–592. 10.1002/jcc.22885 22162017

