# Supplementary material for: Real‐Time Visualisation of Reaction Kinetics and Dynamics: Single‐Molecule Insights into the Iminium‐Catalysed Diels–Alder Reaction
Source: Angew Chem Int Ed Engl. 2025 Sep 9;64(45):e202506535. doi: 10.1002/anie.202506535 (PMC12582012; doi:10.1002/anie.202506535)
Supplement: Supplementary file 1 — Supporting Information [file ANIE-64-e202506535-s002.docx]

Supporting Information for

**Real-Time Visualization of Reaction Kinetics and Dynamics: Single-Molecule Insights into the Iminium-Catalysed Diels-Alder Reaction**

Minsoo Park^[a] †^, Yongdeok Ahn^[a],[b] †^_,_ Juhyeong Cho^[a]^, Juhee Jang^[a]^, Wonhee J. Lee^[a]^, Sangwon Seo*^[a]^, Sunggi Lee*^[a]^, and Daeha Seo*^[a],[b]^

[a] M. Park, Y. Ahn, J. Cho, J. Jang, W. Lee, S. Seo, S. Lee, D. Seo
Department of Physics and Chemistry, DGIST
Daegu 42988, Republic of Korea
E-mail: sangwon.seo@dgist.ac.kr; sunggi.lee@dgist.ac.kr; [livewire@dgist.ac.kr](mailto:livewire@dgist.ac.kr)

[b] Y. Ahn, D. Seo
Department of Chemistry, Pohang University of Science and Technology (POSTECH)
Pohang 37673, Republic of Korea
E-mail: daehaseo@postech.ac.kr

[^†^] These authors contributed equally to this work.

**CONTENTS**

1. Experimental Section ------------------------------------------------------------------------------------- 2

2. Supporting Figures ---------------------------------------------------------------------------------------- 7

3. Supporting Notes ------------------------------------------------------------------------------------------19

4. NMR Spectra ---------------------------------------------------------------------------------------------- 23

5. Computational Details ----------------------------------------------------------------------------------- 26

6. Supporting Movie Legend ------------------------------------------------------------------------------ 33**1. Experimental Section**

**Materials**

For complex synthetic experiments. Pyrrole (98%, Sigma-Aldrich, 131709), 10-undecenoyl chloride (97%, Sigma-Aldrich, 161667), Bromobenzene (99%, Sigma-Aldrich, B57702), Magnesium for Grignard reactions (99.5%, Sigma-Aldrich, 63035), Tetrahydrofuran (THF, 99.9%, Sigma-Aldrich, 401757), 2,4-dimethylpyrrole (97%, Sigma-Aldrich, 390836), Phosphorus(V)oxychloride (99.9%, Sigma-Aldrich, 262099), Triethylamine (TEA, 99.5%, Sigma-Aldrich, 471283), Boron trifluoride diethyl etherate (Sigma-Aldrich, 175501), Dichloromethane (Extra Pure Grade, Duksan, 573), Allyltriethoxysilane (97%,Sigma-Aldrich, A36301), (1,3-Bis(2,4,6-trimethylphenyl)-2-imidazolidinylidene)dichloro(phenylmethylene)(tricyclohexylphosphine)ruthenium (Sigma-Aldrich, 569747), 3-dimethylaminoacrolein (90%, Sigma-Aldrich, 305839), Dicyclopentadiene (95%, Sigma-Aldrich, 36691), (5S)-(−)-2,2,3-Trimethyl-5-benzyl-4-imidazolidinone monohydrochloride (97%, Sigma-Aldrich, 569763), Pyrrolidine (99%, Sigma-Aldrich, P738093), Molecular sieves (3 Å, Sigma-Aldrich, 208574), Methanol-d_4_ (99.8%, Sigma-Aldrich, 151947), Ethanol (99.8%, Sigma-Aldrich, 24102), Hexane (Extra Pure Grade, Duksan, 4189), Ethyl acetate (Extra Pure Grade, Duksan, 1404)

**Optical probe characterization**

UV-vis spectra were recorded on a diode array spectrophotometer (Agilent, Cary 8454). ^1^H spectra were measured with Fourier-transform nuclear magnetic resonance spectrometry (FT-NMR, Bruker, AVANCE III 400). ^1^H NMR chemical shifts are reported in ppm (δ) relative to residual solvent with the solvent resonance employed as an internal standard (CDCl_3_ δ 7.26 ppm). Data are presented as follows: chemical shift, multiplicity (s=singlet, d=doublet, t=triplet, q=quartet, m=multiplet), coupling constants (Hz) and integration. Analytical thin-layer chromatography (TLC) was carried out on Merck silica 60 (230-400 mesh ASTM). High resolution mass (HRMS) spectral data were obtained by electron impact (EI) ionization technique (magnetic sector-electric sector double focusing mass analyzer).

**Synthesis of BODIPY-α,β-enal**

Magnesium (1.8 g, 75.0 mmol) was stirred with dry THF (60.0 mL) in oven-dried flask under N_2_. Bromobenzene in dry THF (7.88 mL, 75.0 mmol, in 15.0 mL) was added dropwise carefully to the solution. When the Mg disappear, pyrrole (5.0 g, 75.0 mmol) was added and the solution was stirred for 3 h under dark at room temperature. 10-undecenoyl chloride (6.4 mL, 30.0 mmol) in dry THF (225.0 mL) was added and the solution was stirred overnight. Saturated aqueous NH_4_Cl was used for quenching the reaction. The mixture was extracted with ethyl acetate. The organic layer was washed with distilled water, dried over sodium sulfate and filtered. The solvent was concentrated in vacuo. The resulting residue was purified by silica gel chromatography (10.0% ethyl acetate in hexanes; R_f_ = 0.3). The pale brown oil was yielded by solvent evaporation and in vacuo (4.41 g, 63.0%). ^1^H NMR (400 MHz, CDCl_3_) δ 10.56 (s, 1H), 7.06 (d, J = 3.1 Hz, 1H), 6.94 (d, J = 3.4 Hz, 1H), 6.26 (q, J = 2.7 Hz, 1H), 5.82 (ddt, J = 16.9, 10.2, 6.6 Hz, 1H), 4.97 (m, 2H), 2.79 (t, J = 7.5 Hz, 2H), 2.05 (q, J = 7.0 Hz, 2H), 1.75 (p, J = 7.4 Hz, 2H), 1.34 (m, 10H). HRMS (EI) m/z: [M]^+^: calcd. for. C_15_H_23_NO 233.1780; Found 233.1782.

**1** (4.1 g, 17.6 mmol) and 2,4-dimethylpyrrole (1.7 g, 17.6 mmol) were dissolved in DCM under magnetic stirring. This mixture was cooled to 0 °C under an inert atmosphere, then phosphoryl chloride (3.4 mL, 36.9 mmol) was added dropwise carefully. After 1 hour, the reaction mixture stirred at room temperature overnight. Sufficient water was added carefully and the organic phase was washed and dried over magnesium sulfate. The solvent was eliminated under reduced pressure. The intermediate was dried in vacuo at 50°C overnight. This intermediate was dissolved in dry DCM in an oven-dried flask. Triethylamine (15.9 mL, 114.0 mmol) was added under inert atmosphere. Then, boron trifluoride diethyl etherate (19.5 mL, 158.0 mmol) was added carefully and the reaction mixture was stirred at room temperature for 6 hours. This reaction was quenched by sufficient water and the organic phase was washed and dried over magnesium sulfate. The solvent was removed under reduced pressure. The purified product was obtained by silica gel chromatography (15.0% ethyl acetate in hexanes; R_f_ = 0.4). The brown oil was isolated by solvent evaporation and in vacuo (2.7 g, 43.4%). ^1^H NMR (400 MHz, CDCl_3_) δ 7.52 (s, 1H), 6.98 (d, J = 4.0 Hz, 1H), 6.36 (dd, J = 4.0, 2.1 Hz, 1H), 6.09 (s, 1H), 5.80 (m, 1H), 4.97 (m, 2H), 2.91 (m, 2H), 2.57 (s, 3H), 2.43 (s, 3H), 1.97 (d, J = 7.1 Hz, 2H), 1.61 (td, J = 7.4, 6.4, 3.7 Hz, 2H), 1.54 – 1.09 (m, 10H). HRMS (EI) m/z: [M]^+^: calcd. for. C_21_H_29_BF_2_N_2_ 358.2392; Found 358.2389.

The Vilsmeier reagent (POCl_3_, 21.2 mmol) dropwise to a solution of 3-dimethylaminoarolein (2.1 mL, 21.2 mmol) in CH_2_Cl_2_ (30.0 mL) at 0 °C under N_2_ for 30 mins. After warming to room temperature, the reaction mixture was further stirred for 1 hour. Then, BODIPY **2** (1.1 mmol) in CH_2_Cl_2_ (45.0 mL) was added to the reaction mixture. After raising the temperature to 40 °C, the reaction mixture was further stirred overnight at which point TLC analysis (5% ethyl acetate in hexanes; R_f_ 0.3) revealed. The reaction mixture was cooled to room temperature. A saturated solution of sodium bicarbonate (50.0 mL) was then added to the crude mixture. The reaction mixture was further stirred for 2 hours and extracted with CH_2_Cl_2_. The organic layers were combined, washed with water, dried with sodium sulfate, and the solvent was removed under reduced pressure. The purified product was obtained by silica gel chromatography (5.0% ethyl acetate in hexanes; R_f_ = 0.2). The red oil was isolated by solvent evaporation and in vacuo (65.6 mg, 15.0 %). ^1^H NMR (400 MHz, CDCl_3_) δ 9.72 (d, J = 7.9 Hz, 1H), 7.89 (d, J = 15.9 Hz, 1H), 7.03 (d, J = 4.3 Hz, 1H), 6.87 (d, J = 4.3 Hz, 1H), 6.66 (dd, J = 15.9, 8.0 Hz, 1H), 6.26 (s, 1H), 5.79 (m, 1H), 4.97 (m, 2H), 2.90 (m, 2H), 2.62 (s, 3H), 2.45 (s, 3H). HRMS (EI) m/z: [M]^+^: calcd. for. C_24_H_31_BF_2_N_2_O 412.2498; Found 412.2502.

To a solution of 2-(Methylamino)ethanol (1.9 mg, 25.1 µmol) in CDCl_3_, **3** (dissolved in 0.5 mL of CDCl_3_) was added dropwise under N_2_ at ambient temperature. The reaction mixture was stirred for 1 day to obtain the N,O-acetal. ^1^H NMR (400 MHz, CDCl_3_) δ 7.15 (d, J = 15.9 Hz, 1H), 7.03 (d, J = 4.0 Hz, 1H), 6.71 (d, J = 4.0 Hz, 1H), 6.22 (dd, J = 15.8, 7.8 Hz, 1H), 6.12 (s, 1H), 6.26 (s, 1H), 5.80 (m, 1H), 4.96 (m, 2H), 4.34 (d, J = 3.8 Hz, 1H), 2.90 (m, 2H), 2.63 (s, 3H). HRMS (EI) m/z: [M]^+^: calcd. for. C_27_H_38_BF_2_ N_3_O 469.3076; Found 469.3071.

The Grubbs catalyst (853.0 µg, 1.0 µmol) was prepared under N_2_ with CH_2_Cl_2_ and a stirring bar in an oven-dried flask. **3** (20.8 mg ,50.3 µmol) and allyltriethoxysilane (56.8 µl,251.0 µmol) were added simultaneously to a stirring solution. The reaction mixture was further stirred overnight at which point TLC analysis (1% ethyl acetate in hexanes R_f_ = 0.2). The solution was directly loaded to silica gel column and purified by ethyl acetate in hexanes. The pale brown oil was isolated by solvent evaporation and in vacuo (15.1 mg, 51%). ^1^H NMR (400 MHz, CDCl_3_) δ 9.72 (d, J = 8.0 Hz, 1H), 7.89 (d, J = 15.9 Hz, 1H), 7.03 (d, J = 4.3 Hz, 1H), 6.88 (d, J = 4.3 Hz, 1H), 6.66 (dd, J = 15.9, 8.0 Hz, 1H), 6.26 (s, 1H), 5.39 (m, 2H), 3.82 (qd, J = 7.0, 1.7 Hz, 6H), 2.91 (m, 2H), 2.63 (s, 3H), 2.45 (s, 3H). HRMS (EI) m/z: [M]^+^: calcd. for. C_31_H_47_BF_2_ N_2_O_4_Si 588.3366; Found 588.3364.

A solution of **3** (37.35 mg ,90.58 µmol) and cat_Mac_ (2.31 mg, 9.06 µmol) were prepared in ethanol under magnetic stirring. Cyclopentadiene (37.32 µL, 0.45 mmol) was then added to the stirring mixture, The reaction was allowed to proceed at room temperature overnight. The solvent was eliminated under reduced pressure. The purified product was obtained by silica gel chromatography (10% ethyl acetate in hexanes; R_f_ = 0.6; 1.4/1.0 *endo*:*exo*). The brown oil was isolated by solvent evaporation and in vacuo (43.26 mg ,88.77 µmol). ^1^H NMR (400 MHz, CDCl_3_) δ 9.88 (d, *J* = 2.4 Hz, 1H), 9.47 (d, *J* = 3.3 Hz, 1H), 7.05 (d, *J* = 4.3 Hz, 1H), 6.95 (d, *J* = 4.2 Hz, 1H), 6.47 (dd, *J* = 5.7, 3.3 Hz, 1H), 6.41 (dd, *J* = 5.8, 3.2 Hz, 1H), 6.34 (d, *J* = 4.2 Hz, 1H), 6.17 (dd, *J* = 5.8, 2.7 Hz, H), 6.12 (s, 2H), 6.04 (d, *J* = 4.2 Hz, 1H), 5.80 (ddt, *J* = 16.9, 10.4, 6.7 Hz, 2H), 4.96 (dd, *J* = 23.6, 13.6 Hz, 4H), 3.59 (d, *J* = 4.9 Hz, 1H), 3.43 (s, 1H), 3.30 (s, 1H), 3.24 (s, 1H), 3.18 (s, 1H), 2.97 (d, *J* = 4.2 Hz, H), 2.85 (q, *J* = 8.4 Hz, 4H), 2.58 (s, 6H), 2.40 (s, 6H). HRMS (EI) m/z: [M]^+^: calcd. for. C_29_H_37_BF_2_ N_2_O 478.2967; Found 478.2962.

**Construction of the flow chamber**

**
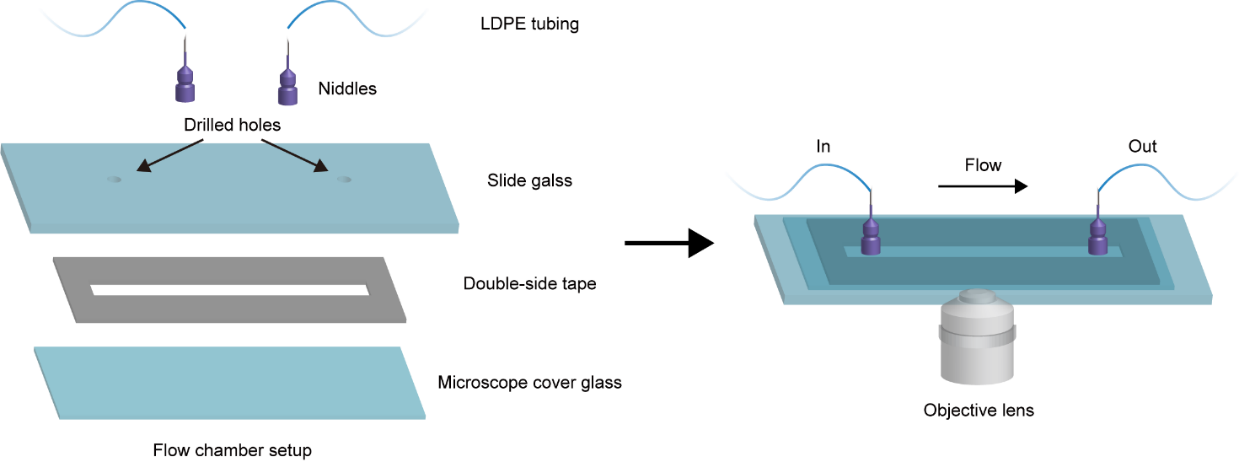
**

Microscope slides with a thickness of 1 mm (76×26 mm, Marienfeld, K08159211) and cover glasses with a thickness of 0.13 mm (24×60 mm, Marienfeld, 0101244) were sonicated in 20 mL of a 50% (1:1) solution of anhydrous acetone and ethanol for 1 hour. After sonication, they were rinsed five times each with deionized water (DI water, 18.2 MΩ) and spectrophotometric grade ethanol. The coverslips and slide glasses were first dried with compressed air then further dried in an oven at 100°C for 30 min. The dried coverslips and slide glasses were treated with piranha solution, composed of 50% (v/v) sulfuric acid (3 parts) and 30% (w/v) hydrogen peroxide (1 part) for 20 min in a fume hood. This solution, known for its strong reactivity with organic substances, was handled with care, using acid-resistant gloves and protective clothing. After treatment, the coverslips were rinsed with DI water and dried under nitrogen gas. After drying, the coverslips and slide glasses were immediately used.

To fabricate a flow chamber, two inlet/outlet holes were drilled through the microscope slide using a precision drill press. The hole spacing was fixed at 45 mm, and the hole diameter was adjusted to be slightly smaller than the outer diameter of the insertion needle (17G, 38 mm; Koreavaccine, 1154998027) to ensure a snug fit. A double-sided adhesive spacer tape (0.16 mm thickness, 3M 467MP-1-5) was applied to the slide glass to define the microfluidic channel geometry, as shown above the figure. A clean coverslip was carefully aligned and placed on top of the tape to seal the chamber. To ensure leak-proof sealing of the channel, epoxy adhesive (JB Weld, K0007841455) was applied around the interface between the coverslip and the slide glass, excluding the drilled holes. Additional epoxy was then applied around each hole to affix the needles, taking care not to clog the internal bore of the needles during curing. After curing the epoxy for 12 hours at room temperature, LPDE tubing (inner diameter 0.58 mm, outer diameter 0.96 mm; Fisher Scientific, 12665497) with a length of 15–20 cm was inserted into each needle. The tubing was gently pushed and pulled within the needle while applying epoxy near the base (approximately 2–5 mm from the tubing end), ensuring firm fixation without occluding the fluidic path. To complete the setup, a syringe was connected to one end of the tubing and sealed at the junction with epoxy. The opposite tubing end was sealed with parafilm to prevent backflow or evaporation. After 12 hours of additional curing, the chamber was primed by submerging the tubing into the desired solution and gently pulling the plunger of the syringe to draw liquid into the flow channel. The assembled chamber allowed for the controlled flow of reagents and real-time observation of reactions within the microfluidic environment.

**Observation of chemical reaction at single-molecule level**

A flow chamber was assembled with a pre**-**cleaned coverslip and employed to monitor the reaction under continuous flow conditions. An ethanol solution of BODIPY-α,β-enal (10 nM) was flowed, resulting in the immobilization of approximately 20-50 individual molecules within a 1.77×10^4^ µm^2^ imaging area. Subsequently, cat_Mac_ solutions (7.5-15 mM) were added into the chamber. For the Diels-Alder reaction imaging, a 7.5 mM cat_Mac_ solution was first loaded, followed by diene solutions at concentrations (5.9-11.9 mM).

Single-molecule experiments were performed using total internal reflection fluorescence (TIRF) microscope. The TIRF microscope is equipped with a laser (Nikon, LU-N4 Laser Unit, 488 nm) to **S** at its respective excitation wavelength**,** a perfect focus system (PFS, TI2-N-ND-P), a motorized stage, an electron multiplying charge-coupled device (EMCCD, Andor, iXorn Ultra 897) at its respective excitation wavelength. An oil-immersion lens (Nikon, 1.49 NA, oil-immersion, CFI SR HP Apochromat TIRF) was used for detection of single dye.

**Determining the number of states in time trajectory at the single molecule level**

The adaptability of the HMM framework allows for the application of various model types to the data. This method is most suitable when using the simplest model with the smallest number of free parameters that match the data. To determine the optimal number of states, The Bayesian Information Criterion (BIC) is performed in trajectory analysis of single molecule, which is a widely used method with a theoretical basis and computational accessibility. BIC, which serves as a general standard to find the most likely number of states (n), is formally defined as shown below.

$$BIC=k ln\left( N \right)-2ln(\hat{L})$$

where L is the maximum value of likelihood function, k is the number of free parameters being fit, including transition rates, average signa values, and standard deviation, and N is the number of measured data points. By using Baum-Welch algorithm for maximum likelihood estimation, BIC as a function of the number of states determines the true model by finding the minimum point where the BIC slope abruptly decreases before increasing.

The best fit among various models is indicated by the lowest BIC score in the comparison, and this contributes to maximize data likelihood and minimize model complexity. The BIC plot in S1 reveals the optimal number of states in time-binned data of single organic dye. The 2-state fits of starting material and 4-state fits of chemical reaction with secondary amine catalyst have the lowest BIC, while all other models exhibit the expected trend of rising BIC with an increasing number of free parameters. The separation of intensity trajectory by HMM analysis is verified by examination of BIC, which determines optimal number of states showing specific chemical species in chemical reaction.

**2. Supporting Figures**


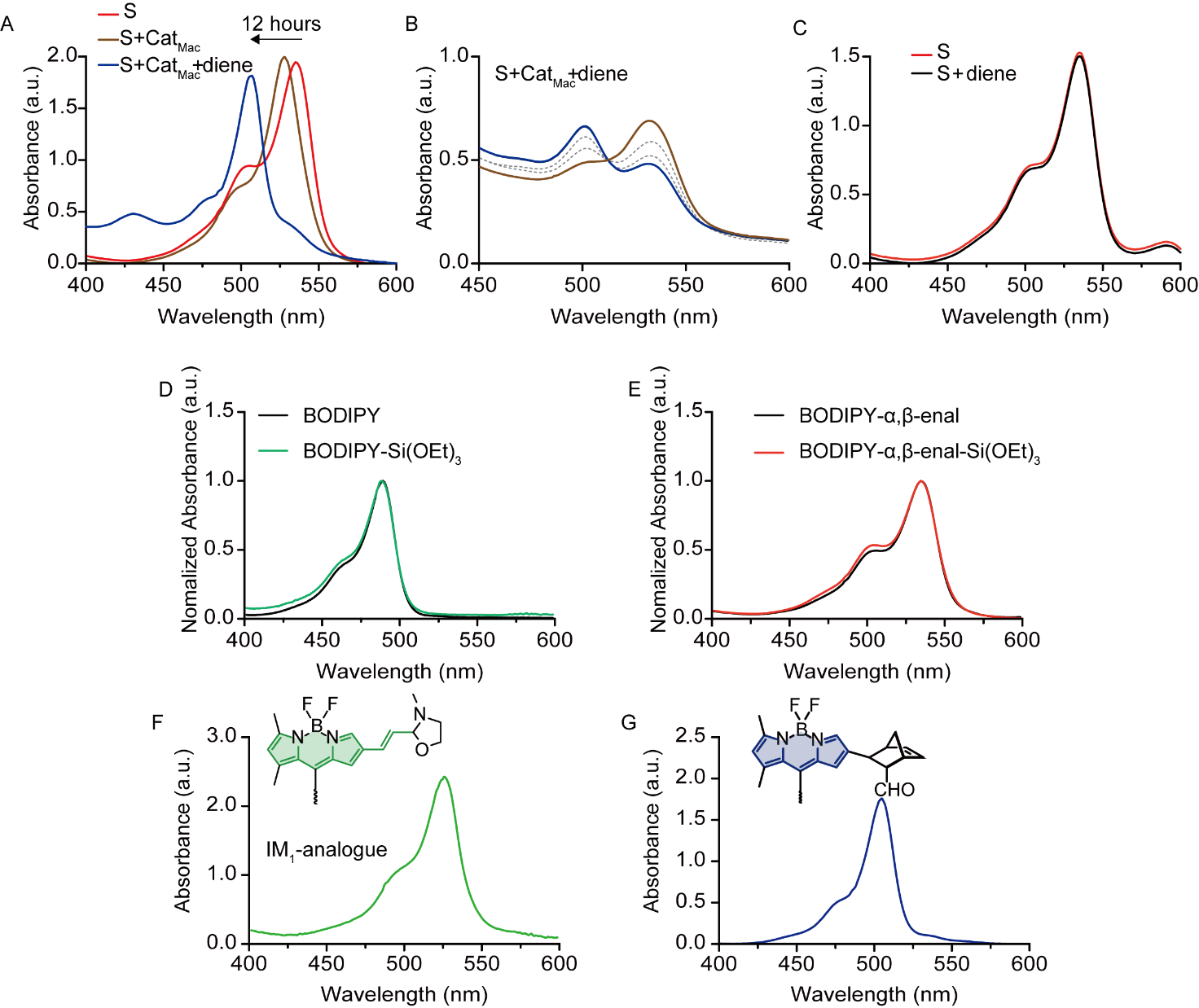


**Figure S1.** Characterization of designed optical probes and chemical reaction in ensemble measurement**.** (A) The absorption spectra of Diels-Alder reaction with excess cat_Mac_ and diene in ethanol over 12 hours. The reaction of **S** (red line) with cat_Mac_ showed a spectral shift to mixture of reactants (brown line). Addition of diene to the reaction mixture resulted in the formation of the product (blue line) and followed as a shift in the absorption spectrum. (B) UV-vis spectral changes of **S**+cat_Mac_ upon addition of diene in ethanol. The presence of isosbestic point suggested the absence of irreversible side reactions, providing evidence for the validity of the reaction mechanism. (C) Diels-Alder reaction of **S** with only diene. The result showed no difference over 12 hours, indicating that the reaction in this designed system required cat_Mac_ to proceed. (D) UV/Vis absorption spectra of BODPY and BODIPY-Si(OEt)_3_. (E) The absorption peak of BODIPY-α,β-enal and BODIPY-α,β-enal-Si(OEt)_3_. Alkoxysilane group does not affect the optical properties. (F,G) The absorption spectra of chemical species similar to IM_1_ and final product of whole process.


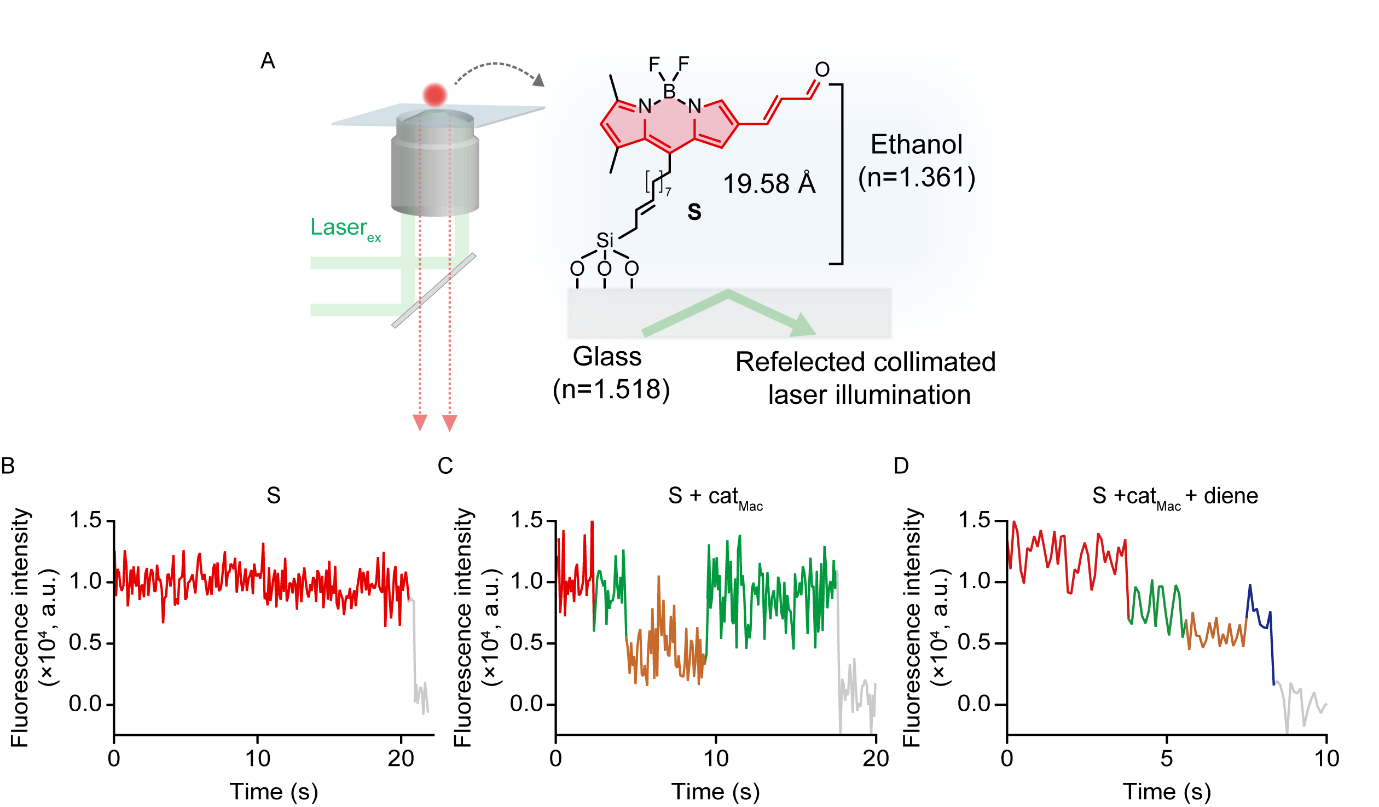


**Figure S2.** Experimental design for observing chemical reactions at the single-molecule level using total internal reflection fluorescence microscopy. (A) The design of **S** incorporating a long alkyl chain siloxyl group for single-molecule imaging. The functionalized probe, with a molecular size of 19.58 Å, is optimal to minimize steric hindrance between the probe and the glass surface. This experimental setup allowed for sub-second temporal resolution (~100 ms) and extended observation periods of up to 1 minute without photo-blinking. (B,C,D) Time traces of individual probes measured under three different conditions based on the addition of the cat_Mac_ and diene-absence of both, presence of the cat_Mac_ alone, and presence of both the cat_Mac_ and diene. The intensity trajectories were tracked for 1 minute and analyzed using the Hidden Markov Model.


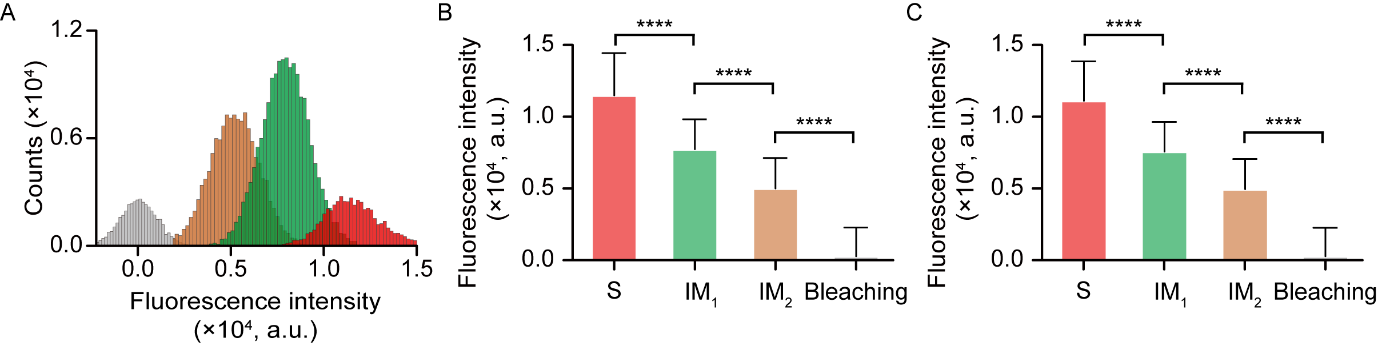


**Figure S3.** Fluorescence intensity-based validation of the HMM-classified states. (A) Fluorescence intensity histograms of the four distinct states identified by HMM analysis (**S**, **IM_1_,** **IM_2_**, and the photobleached state) at a catalyst concentration of 15.0 mM. (B, C) Statistical comparison of the mean fluorescence intensities across the various states reveals significant differences among all pairs (****p < 0.0001, one-way ANOVA with Tukey’s post-hoc test). The results shown are representative and reproducible across three independent experimental sessions.


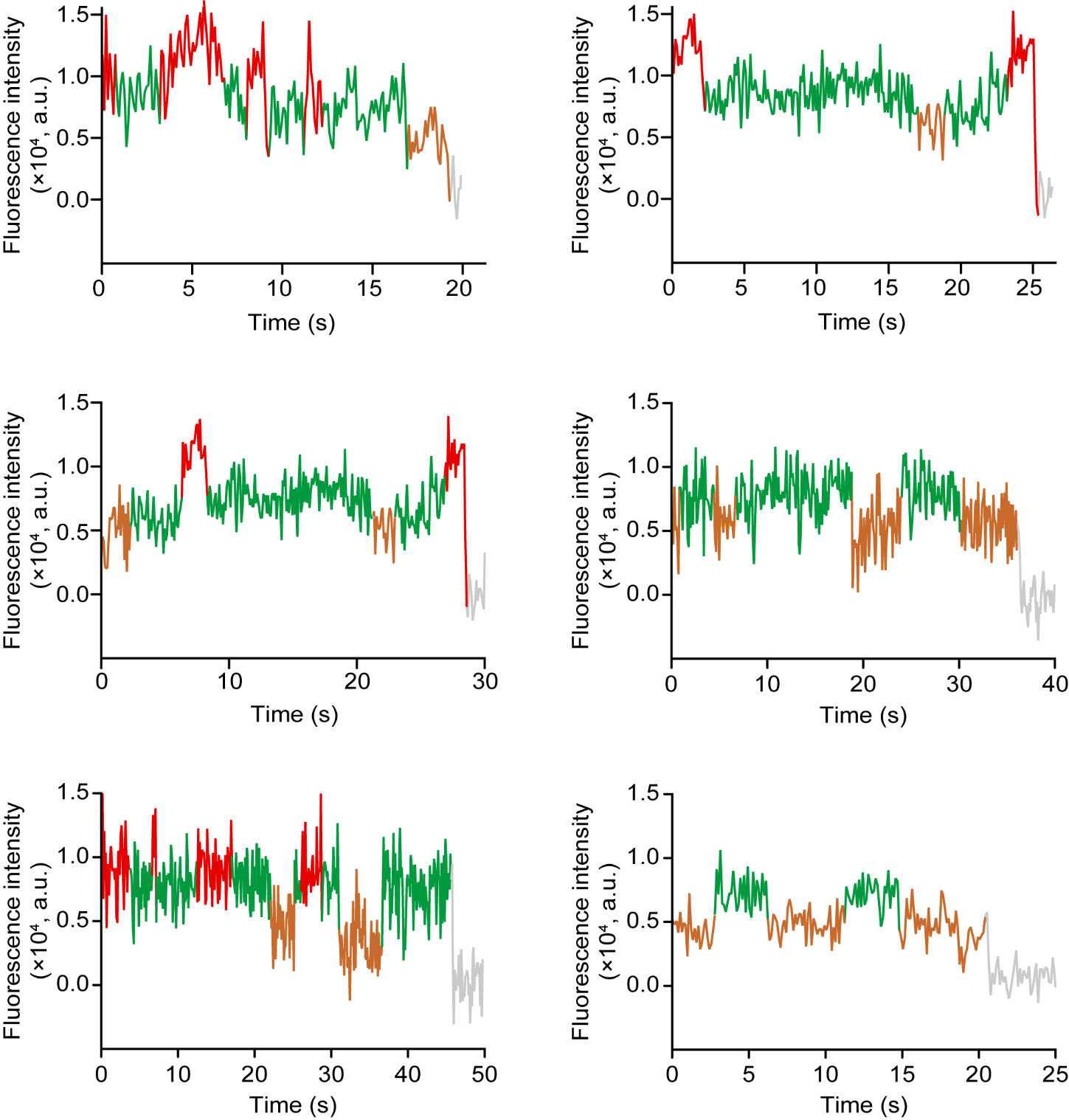
**Figure S4.** HMM-based separation of fluorescence intensity states in a chemical reaction catalysed by the MacMillan catalyst. Representative intensity trajectories showing the **S** (red), **IM_1_** (green), **IM_2_** (brown), and bleaching (grey) states under [cat_Mac_] = 15.0 mM conditions.


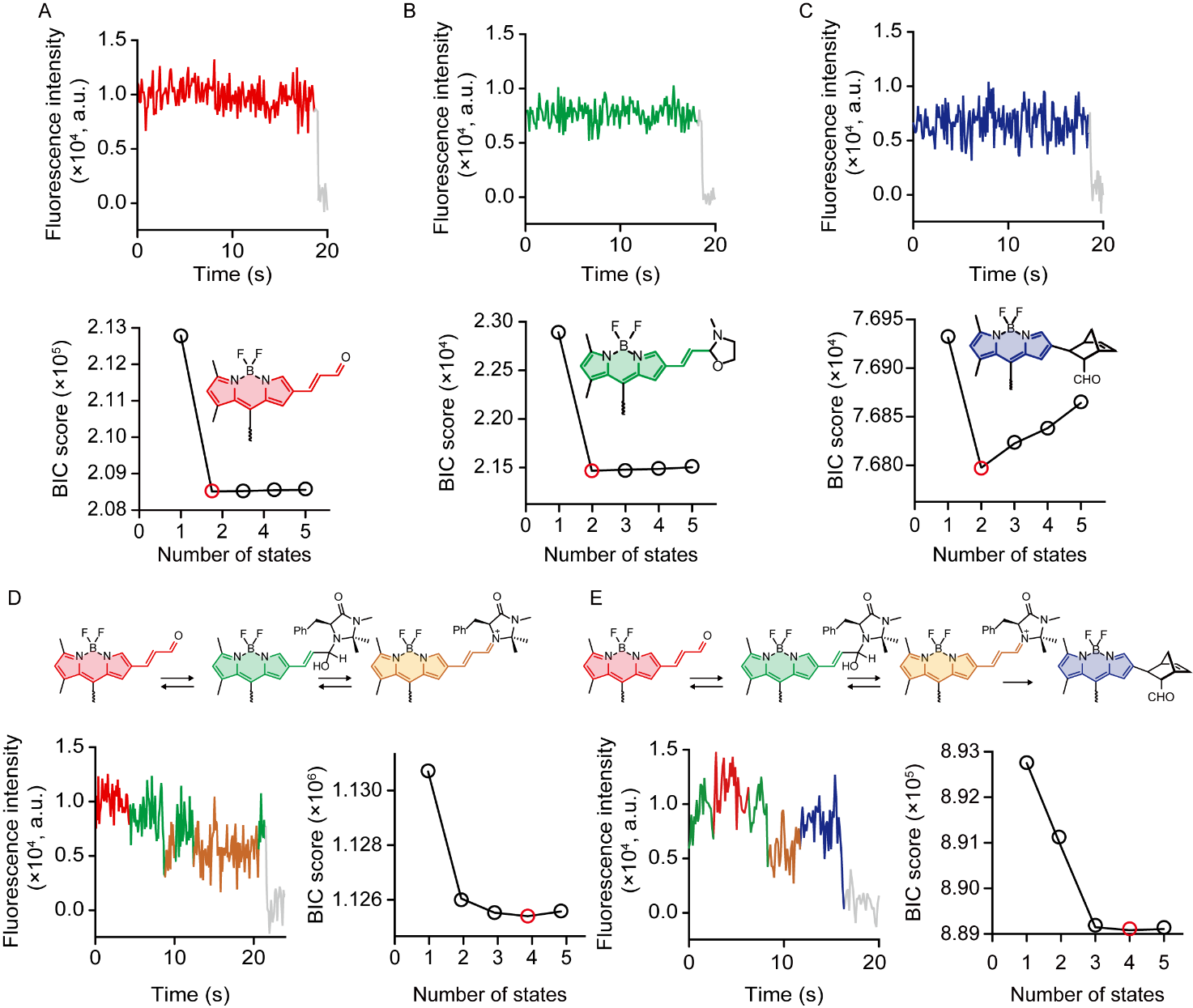


**Figure S5.** Determination of the optimal number of hidden states from intensity time series. The Bayesian information criterion (BIC) is employed to deduce the simplest model with the smallest number of free parameters that match the data. (A,B,C) Intensity trajectories of starting material, **IM_1_**-analogue and product, along with their number of states in HMM model versus BIC scores. The 2-state fits of optical probes and have the lowest BIC, while all other models exhibit the expected trend of rising BIC with an increasing number of free parameters. (D,E) Intensity trajectories and BIC scores for chemical reactions using cat_Mac_ alone and cat_Mac_ with diene. In chemical reactions, the 4-state model was identified as the best fit based on the lowest BIC score, where the slope showed an abrupt decrease before increasing.

**
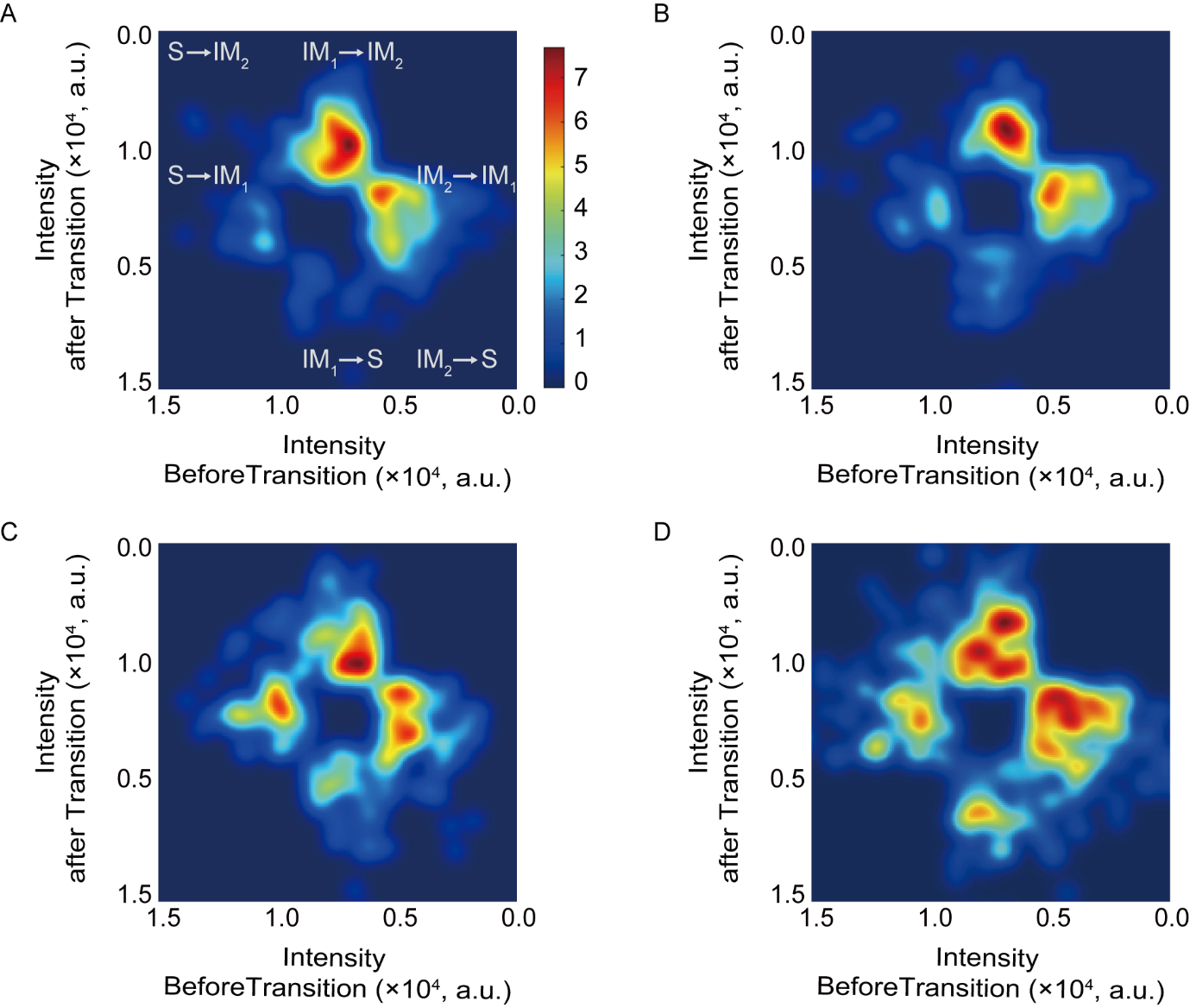
**

**Figure S6.** Transition density plots of the cumulative transition intensities in iminium ion formation process using HMM method. After collecting hundreds of fit traces during chemical reaction with secondary amine catalyst (A) 7.5, (B) 10.0, (C) 12.5, (D) 15.0 mM. The graph represents the average intensity before a transition on the x-axis and after the transition on the y-axis. The transition density plots indicate transitions occurring predominantly between neighboring intensity values, which suggested that starting material undergoes a reaction to form intermediates in sequence such as proposed mechanism. In experiments with varying [cat_Mac_], Transitions from S to IM_2_ and from IM_2_ to S are virtually undetected. The scale bar represents the relative density of state-to-state transitions, with blue indicating low-density regions and red denoting high-density regions along the gradient.

**
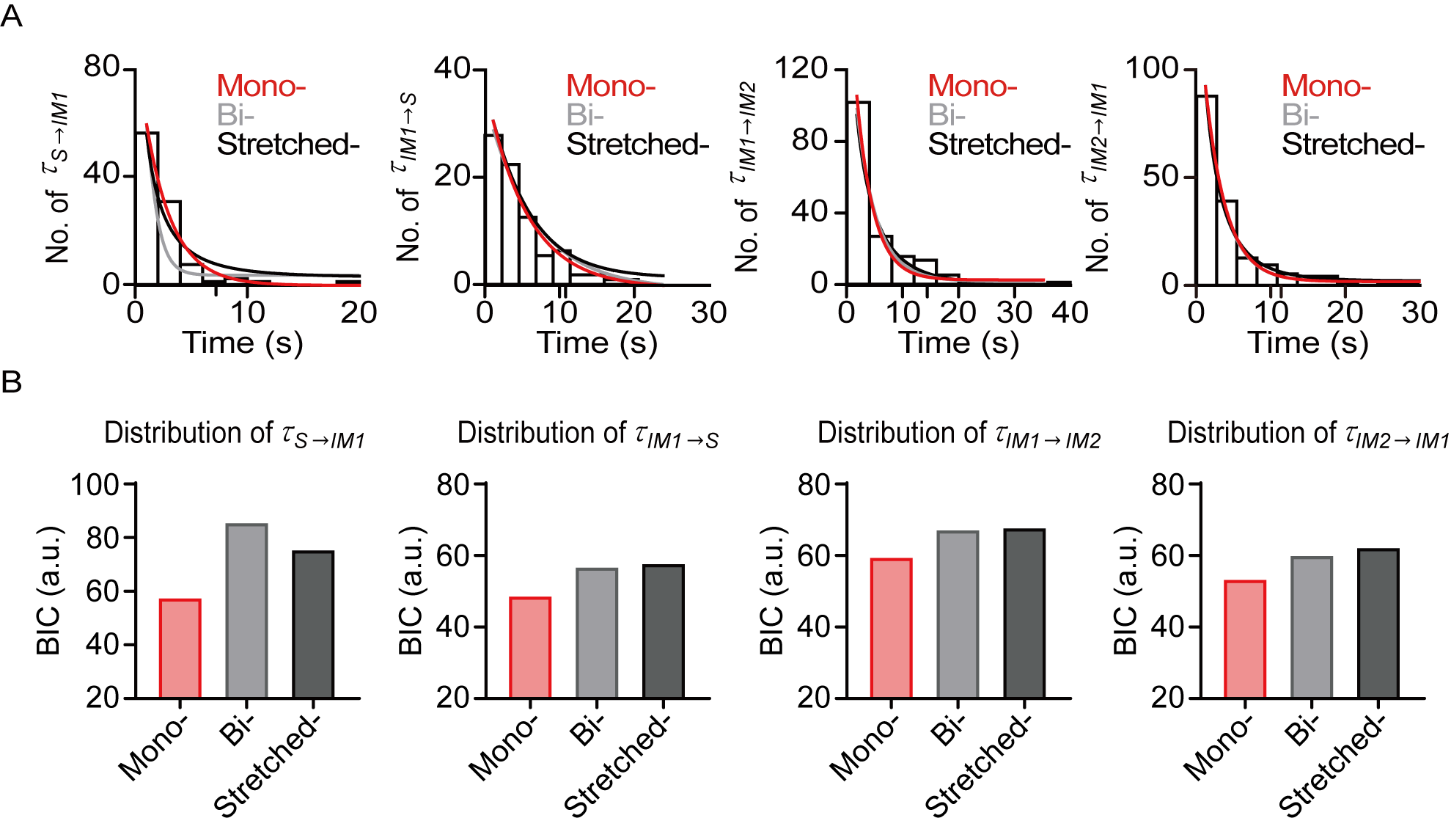
**

**Figure S7.** Model comparison of the dwell time distributions across four elementary transitions. (A) Dwell time distributions for each elementary transition were fitted using mono-exponential, bi-exponential, and stretched-exponential models. (B) Corresponding BIC values for the mono-exponential (red), bi-exponential (grey), and stretched-exponential (black) fits. In all cases, the mono-exponential model exhibited the lowest BIC values, indicating that it describes the dwell time distributions more accurately.

**
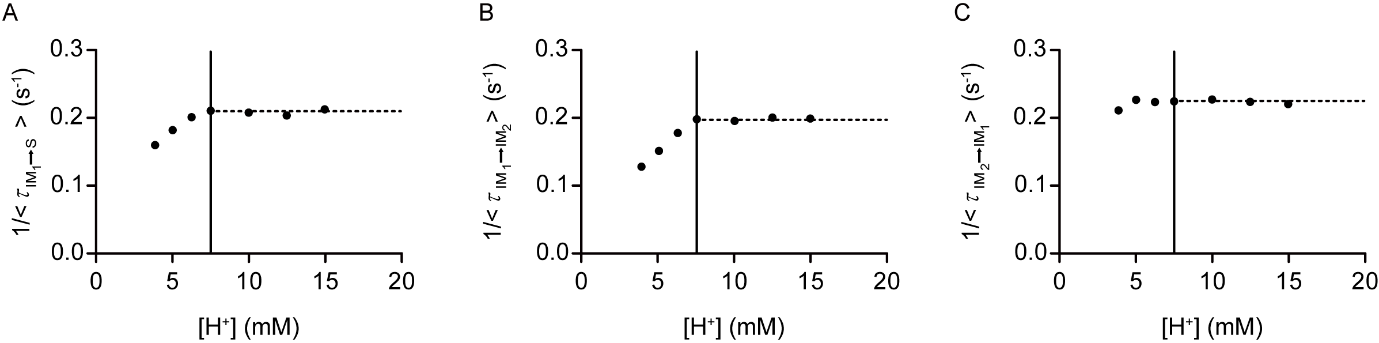
**

**Figure S8.** Concentration dependence of the reversible formation of an iminium ion intermediate with respect to proton concentration. Experiments are in ethanol solution with synthesized non-salt form of cat_Mac_ and hydrochloric acid as a proton source. (A,B,C) The plots of <$\tau_{{IM}_{1}\to S}$>^-1^, <$\tau_{{IM}_{1}\to{IM}_{2}}$>^-1^ and <$\tau_{{IM}_{2}\to{IM}_{1}}$>^-1^ , representing the reciprocals of mean dwell times, at varying proton concentrations. <$\tau_{{IM}_{1}\to S}$>^-1^ and <$\tau_{{IM}_{1}\to{IM}_{2}}$>^-1^ are increased under [proton]= 3.75, 5.00, 6.25 mM. When the [proton] exceed 7.5 mM, these rate constants are independent of it.


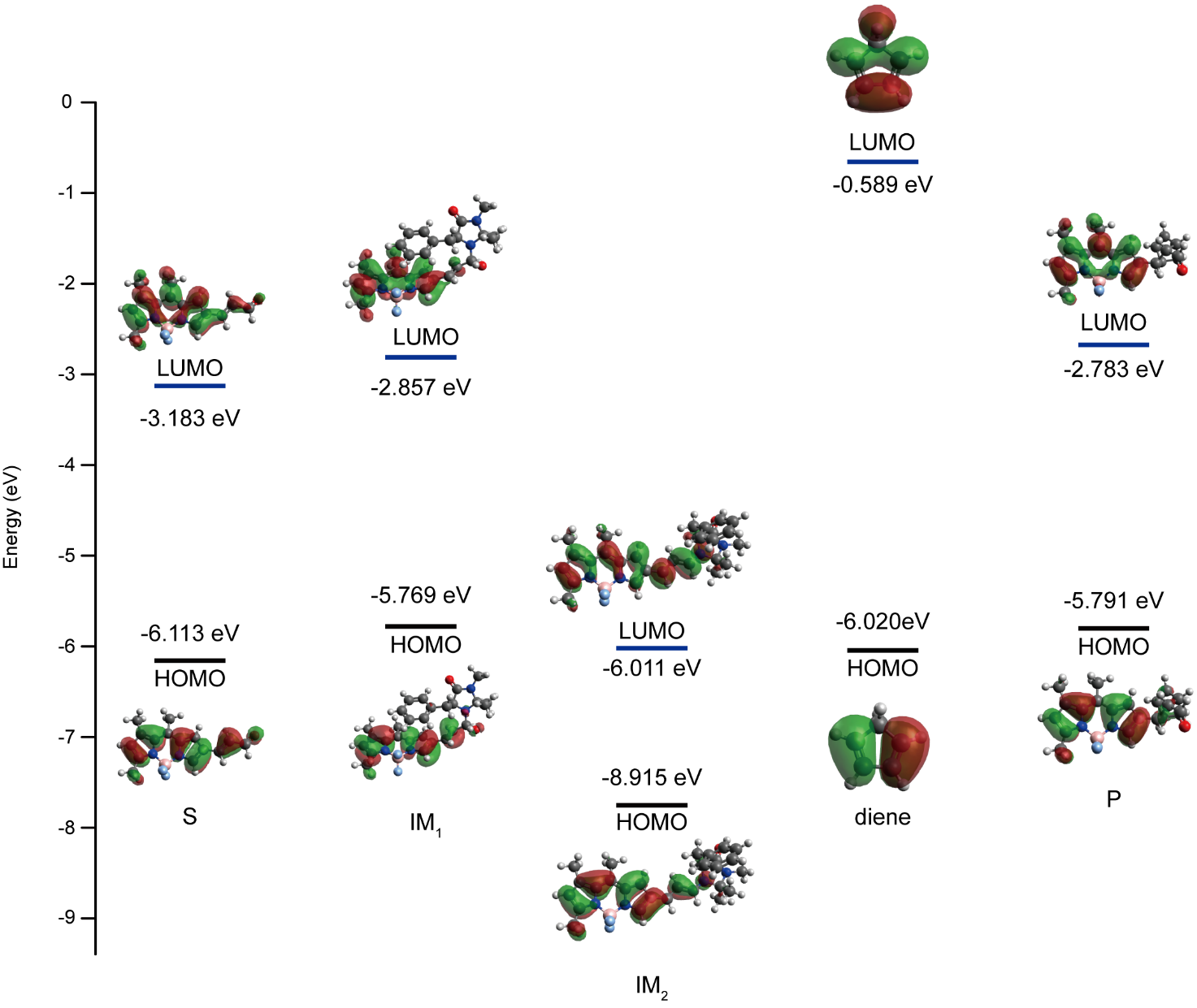
**Figure S9.** Shapes and energies of HOMO (black) and LUMO (green) of BODIPY and its derivatives calculated at the uB3LYP/def2svp level for H,B,C,N,O,F.


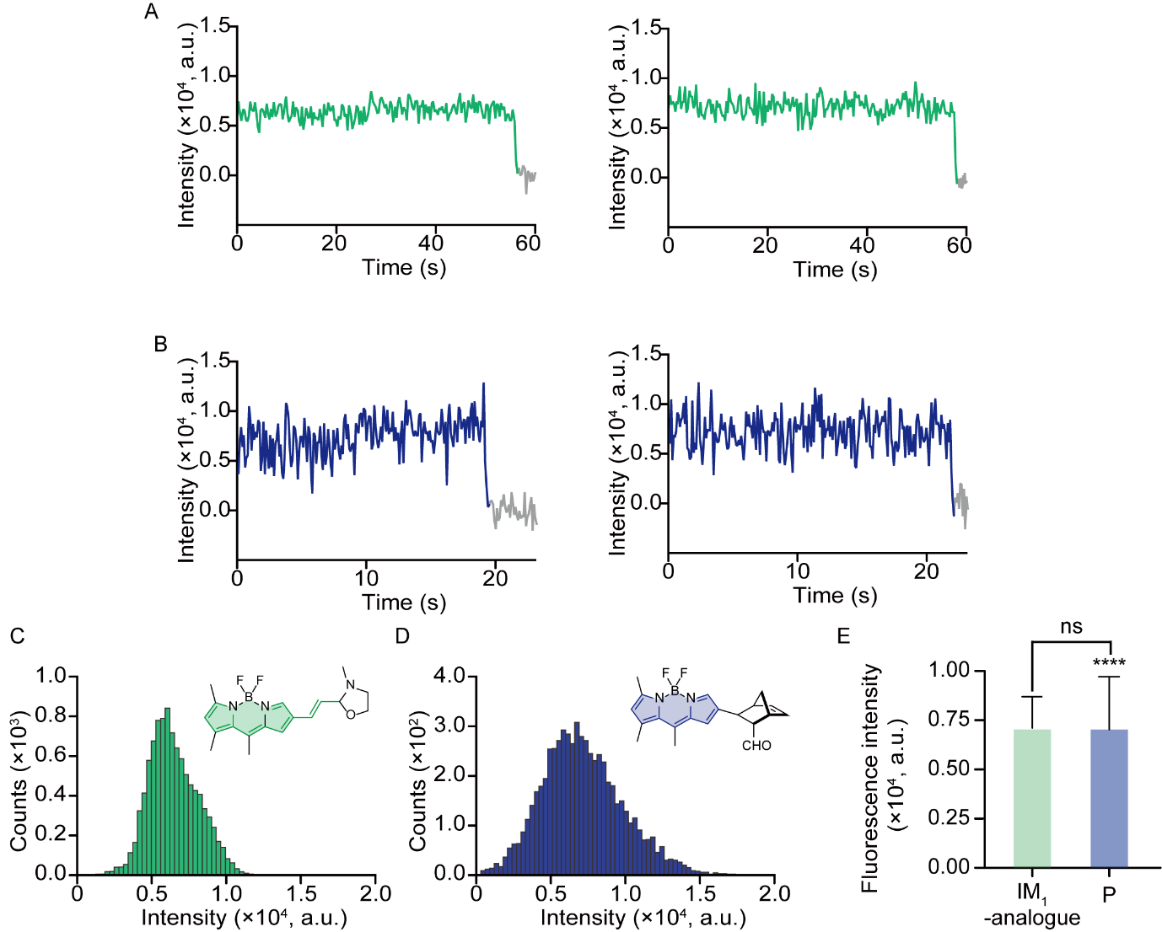


**Figure S10.** Differentiation between the **IM₁**and product (**P**) states based on fluorescence signal variance. (A, B) Representative single-molecule fluorescence trajectories for the **IM₁**analogue (green) and product **P** (blue). Note the significantly higher fluorescence fluctuations observed in the **P** state. (C, D) Fluorescence intensity histograms demonstrating comparable mean intensities between the **IM₁** and **P** states. (E) Although the mean intensities do not differ significantly (n.s., unpaired t-test), variance analysis reveals highly significant differences (****p < 0.0001, F-test), indicating distinct photophysical characteristics between the **IM₁** and **P** states.


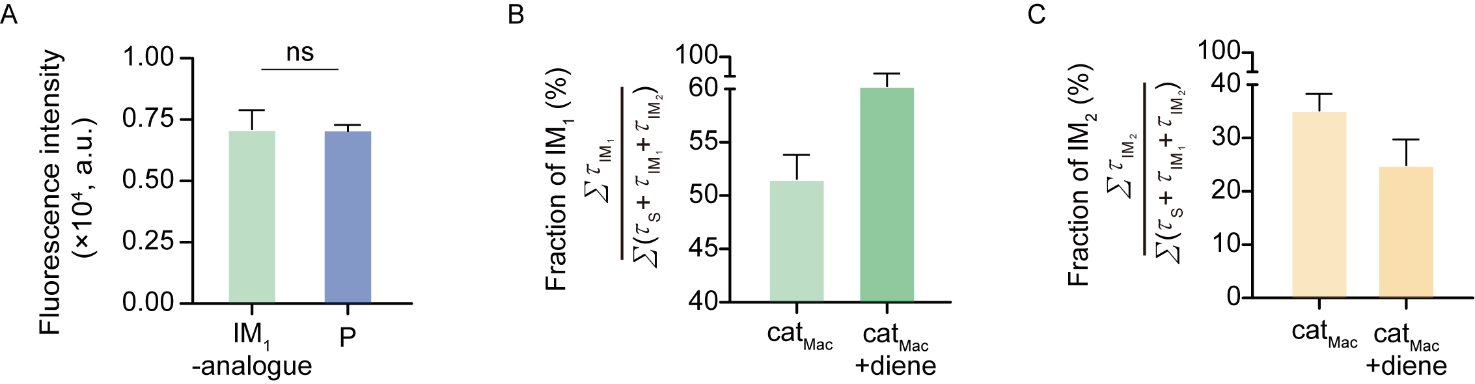
**Figure S11.** Statistical analyses of the fluorescence intensities and dwell times of the various states using cat_Mac_ and the diene. (A) Mean fluorescence intensities for **IM_1_**-anlalogue and **P** observed at the single-molecule level. Statistical analysis showed no significant difference between the emission intensities of the two probes. (B,C) Distributions of the dwell times for **IM_1_** and **IM_2_**. The dwell times of the HMM-estimated **IM_1_** and **IM_2_** states were measured under two sets of conditions, namely the addition of cat_Mac_ alone, and the addition of both cat_Mac_ and the diene. The difference in the lifetime of the HMM-estimated **IM_1_** state was attributed to the presence of the **IM_1_** and **P** states during the Diels–Alder reaction. The decrease in the fraction of the **IM_2_** dwell time for the system containing both cat_Mac_ and the diene results from its participation in the formation of the reaction product.


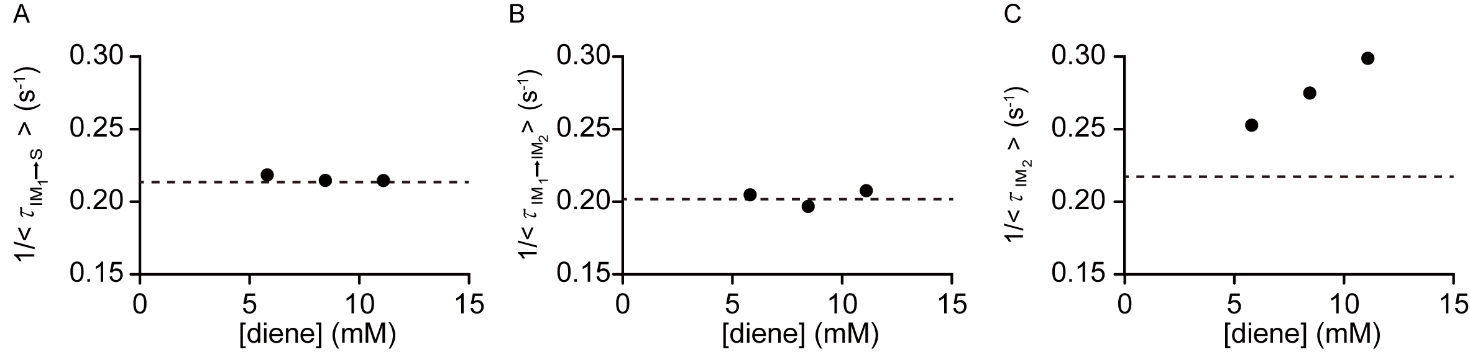


**Figure S12.** Concentration dependence of the Diels-Alder reaction at the single molecule level. (A,B) Plots of the reciprocals of the mean waiting times <$\tau_{{IM}_{1}\to S}$>^-1^ and <$\tau_{{IM}_{1}\to{IM}_{2}}$>^-1^ . The rates showed constant values versus the diene concentration. (C) Diene concentration dependence of <$\tau_{{IM}_{2}}$>^-1^. The reciprocals of the mean dwell times of **IM_2_** showed an increase correlated with [diene]. <$\tau_{{IM}_{2}\to{IM}_{1}}$> and <$\tau_{{IM}_{2}\to P}$> were related to the mean lifetime of **IM_2_** $(<\tau_{{IM}_{2}}>)$ based on $<\tau_{{IM}_{2}}>$=1/($k_{{IM}_{2}\to{IM}_{1}}+k_{{IM}_{2}\to P}$) =$<\tau_{{IM}_{2}\to{IM}_{1}}><\tau_{{IM}_{2}\to P}>/(<\tau_{{IM}_{2}\to{IM}_{1}}>+<\tau_{{IM}_{2}\to P}>).$The dashed line represents the rate constants of each reaction obtained with the addition of cat_Mac_ alone.

**3. Supporting Note**

**Note S1. Trade-offs in Single-Molecule Fluorescence Microscopy: Chemical Identification vs. Temporal Resolution**

Microscopy-based approaches inherently face trade-offs between various experimental parameters. For example, achieving a higher spatial resolution in fluorescence microscopy often comes at the expense of the temporal resolution owing to the requirement for longer photon-collection times. Similarly, in single-molecule chemical mechanistic studies, another important but often overlooked trade-off arises between the temporal resolution and precise chemical identification.

Rigorous chemical identification traditionally relies on spectroscopic approaches, such as nuclear magnetic resonance (NMR), infrared (IR), or fluorescence spectroscopy, because of the distinct and highly informative spectral signatures of these methods. However, acquiring such spectra typically demands high photon budgets and long integration times (i.e., often seconds or even minutes), which far exceed the millisecond-scale temporal resolutions necessary to monitor transient reaction intermediates.

To illustrate this practical constraint within the current experimental context, a dual-path detection system capable of simultaneously capturing single-molecule fluorescence images and spectral data was constructed (see Figure S13). In this system, ~10% of the emitted photons were directed to an electron multiplying charge-coupled device (EMCCD) camera for real-time single-molecule imaging, while the remaining 90% were collected by a spectrometer (Andor Shamrock 193i-A with Newton DU-971 CCD) for spectral analysis. Despite extensive optimisation, significant technical challenges were encountered. More specifically, at the standard temporal resolution (~100 ms/frame) employed in these single-molecule kinetic analyses, the photon yields were insufficient for obtaining reliable single-molecule spectra. Furthermore, attempts to increase the photon yield via intensified excitation resulted in rapid photobleaching, severely disrupting continuous real-time imaging.

Indeed, reliable single-molecule spectra could only be acquired with integration times exceeding 5 s (Figure S13), which are too slow to capture dynamic reaction intermediates and kinetic transitions that occur within milliseconds. This experimental outcome clearly demonstrates the fundamental trade-off between rigorous chemical identification through spectral validation and the ability to monitor real-time molecular kinetics.


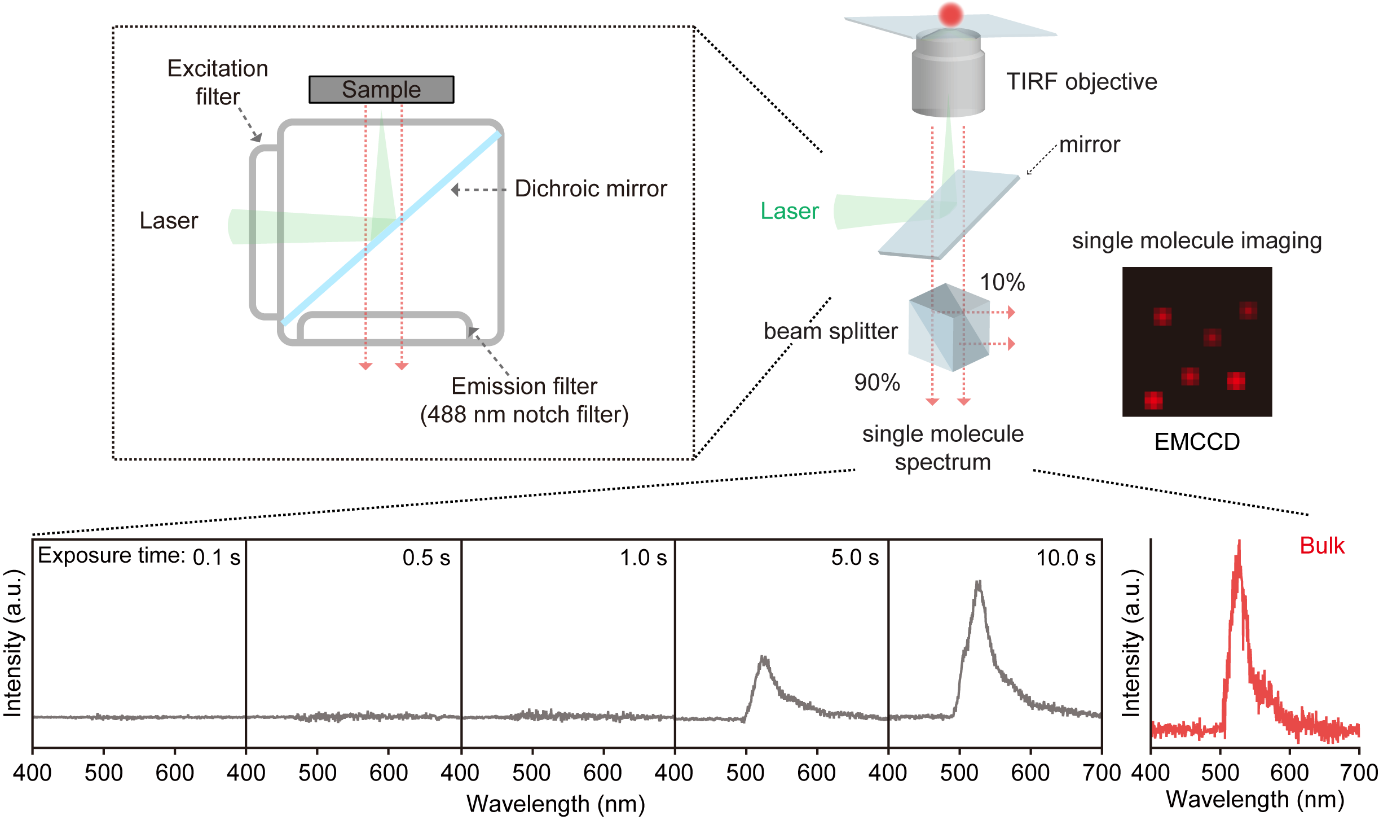


**Figure S13. Schematic illustration of the custom dual-path detection system developed for simultaneous single-molecule fluorescence imaging and spectral acquisition.** Under the optimised imaging conditions (~100 ms exposure), reliable single-molecule spectra were unattainable owing to an insufficient photon yield and rapid photobleaching. Reliable spectral acquisition therefore required significantly extended exposure times (≥5 s) that are incompatible with real-time kinetic analyses.**Note S2. Can Conformational Changes in Single Molecules Be Observed via Optical Microscopy?**

**Background and Hypothesis**. In enantioselective catalysis, understanding the structures of reactive intermediates is critical to elucidating detailed reaction mechanisms, especially the reaction pathways that dictate product formation and selectivity. Among such intermediates, the iminium intermediate (**IM_2_**), which is formed in reactions catalysed by the first-generation MacMillan catalyst (cat_Mac_), is widely accepted to exist predominantly in two distinct conformers, namely one proposed by MacMillan (**IM_2,Mac_**) and another proposed by Houk (**IM_2,Houk_**) (Figure S14A).^[22,58–63]^

The MacMillan conformer (**IM_2,Mac_**) is characterised by a compact geometry in which the phenyl substituent (C_6_H_5_) closely interacts with the iminium ion via cation–π interactions. This arrangement differs markedly from the Houk conformer (**IM_2,Houk_**), which adopts a more open geometry stabilised primarily by CH–π interactions. Computational studies suggest that the energy difference between these conformers is small (~1–2 kcal/mol), and both conformers are likely to coexist at room temperature, albeit in varying population ratios depending on the specific reaction conditions. Experimental evidence from NMR spectroscopy, vibrational circular dichroism, and crystallography consistently supports the predominance of the **IM_2,Houk_** conformer in solution, with reported population ratios ranging from approximately 3:1 to as high as 25:1 in solid-state structures.^[22,58]^

Building on this background, the current study aimed to test the hypothesis that these two conformers exhibit distinct photophysical behaviours that may be detectable at the single-molecule level. Specifically, it was hypothesised that the cation–π interaction characteristics of **IM_2,Mac_** would modulate the π-conjugation efficiency and the electron distribution, causing transient fluorescence quenching. It was therefore postulated that if such conformational changes occur on experimentally observable timescales, they could be indirectly detected via optical fluorescence microscopy through characteristic fluorescence intensity fluctuations.

**Fluorescence quenching by photoinduced electron transfer (PET)**

Fluorescence quenching through interactions between dyes and organic molecules has been widely employed in the field of biotechnology. More specifically, various fluorescent probes, including BODIPY, fluorescein, and rhodamine, have been widely used in biotechnology due to their ability to promote photoinduced electron transfer (PET)-driven quenching via intramolecular functional groups or interactions with nearby nucleobases. Indeed, PET plays a crucial role in modulating fluorescence, and has been extensively applied in sensing and imaging technologies. Generally, PET involving a fluorophore in its first excited singlet state leads to the formation of a radical D/A ion pair (D^+^-A^−^). Upon charge recombination, the system subsequently relaxes to the ground state, resulting in a non-radiative process. The efficiency of charge separation depends on the oxidation and reduction potentials, the reorganisation energy, and the spatial distance between the donor and the acceptor. The efficiency of photoinduced charge separation can be estimated from the change in free energy (∆G_cs_), as described by the Rehm-Weller equation:

$\Delta G_{cs}=E_{ox}-E_{red}-E_{0,0}+C$,

where $E_{ox}$ and $E_{red}$ are the ground state oxidation potential of the donor and the reduction potential of the acceptor, respectively, in the solvent environment. Additionally, $E_{0,0}$ is the energy of the zero-zero transition to the lowest excited singlet state, and C is the solvent dependent Coulombic interaction energy (which can be neglected in a moderately polar environment). ∆G_cs_ values between −0.5 and 0.2 have been reported to be sufficient for fluorescence quenching by PET. Based on the calculated oxidation potential of methylbenzene (2.02 V vs SCE), the calculated reduction potential (−0.70 V vs SCE) of BODIPY, and the $E_{0,0}$ value (2.90 eV) of the BODIPY moiety at the B3LYP/def2svp level of theory, ∆G_cs_ was determined to be −0.18 eV, which enables **IM_2,Mac_** to undergo fluorescence quenching under the given observation conditions.

**Proposed Photophysical Mechanism and Computational Support.** To explore the feasibility of this hypothesis, single-molecule fluorescence microscopy experiments were performed using cat_Mac_ and specifically analysed transient fluorescence darkening (quenching) events. Notably, brief, intermittent quenching exclusively was observed within the **IM_2_** trajectories (Figures S14B and S14C), whereas no similar quenching events occurred in trajectories corresponding to the initial state (**S**) or in control experiments using a simplified catalyst that lacked aromatic and methyl substituents (cat_ctrl_; Figure S9). These results strongly imply that the observed fluorescence quenching events are directly related to specific interactions inherent to the **IM_2,Mac_** conformer.


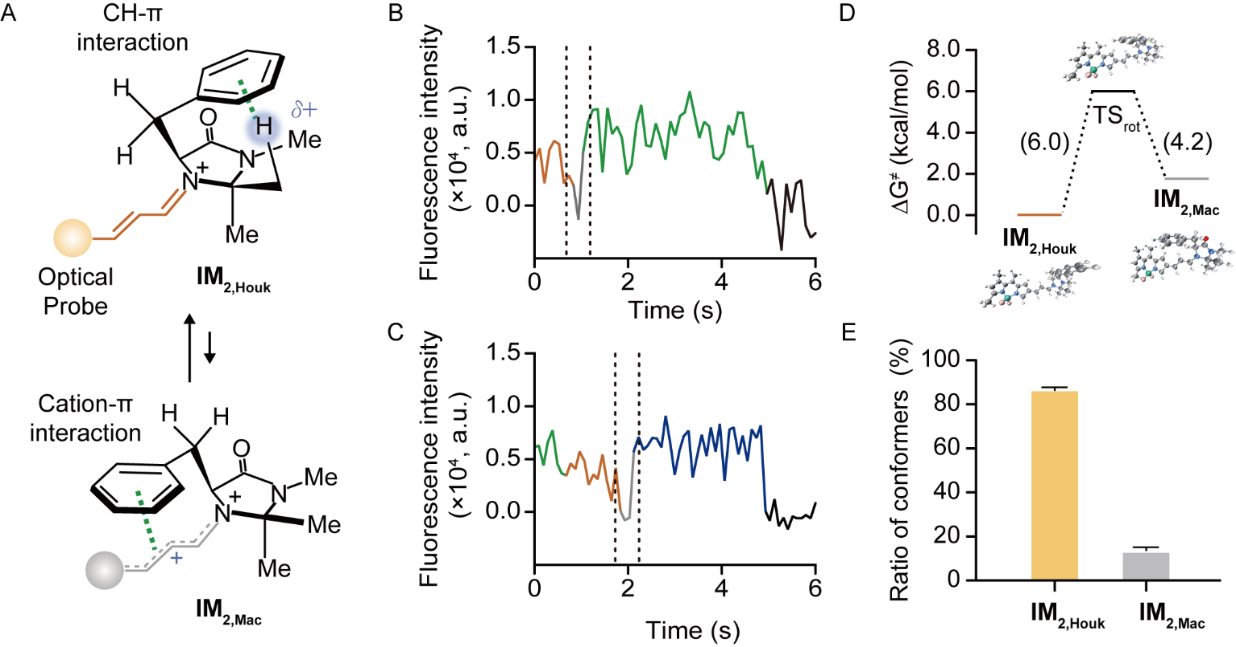


**Figure S14.** Identification of the iminium ion conformers. (A) Intermolecular interactions occurring in the presence of cat_Mac_, showing the two staggered conformers of the MacMillan iminium salt derived from the BODIPY-α,β-enal. (B,C) Variation in the representative fluorescence intensity over time for fluorescence quenching in the presence of cat_Mac_ alone and cat_Mac_ plus the diene. Single-molecule traces recorded for **IM_2,Houk_** (yellow line) and IM_2,Mac_ (grey line), showing that conformational interconversion originated from rotation of the benzyl unit during iminium ion formation. (D) Energy profile for the interconversion between **IM_2,Houk_** and **IM_2,Mac_**, as determined at the uM062X-D3/def2-TZVPP/SMD(EtOH)//uB3LYP-D3/def2-SVP level of theory. (E) Relative abundances of the two iminium conformers. The ratio between **IM_2,Houk_** and **IM_2,Mac_** was determined to be 6.6:1.

The proposed mechanism underlying this transient quenching involves the compact geometry of **IM_2,Mac_**, where cation–π interactions significantly alter the electron distribution within the aromatic ring–iminium system. Quantum chemical calculations supported this photophysical mechanism by predicting a favourable free energy change for charge separation (∆G_cs_), consistent with electron redistribution in the MacMillan conformer. These calculations further substantiate the proposed quenching mechanism as a robust theoretical framework for interpreting our experimental observations.

**Temporal Resolution and Experimental Limitations.** Despite these promising indirect observations, critical limitations remain regarding temporal resolution in single-molecule optical experiments. For instance, computational calculations suggest an extremely low rotational barrier (~6.0 kcal/mol) between the **IM_2,Houk_** and **IM_2,Mac_** conformers, indicating that conformational interconversion is expected to occur extremely rapidly at room temperature (i.e., on the microsecond or even nanosecond timescale) (Figure S14D).^[58,60]^ The current fluorescence microscopy approach employs a temporal resolution of ~100 milliseconds per imaging frame, which is several orders of magnitude slower than the predicted interconversion rates.

Because of this intrinsic timescale mismatch, the direct and continuous tracking of conformational changes between the two **IM_2_** conformers remains beyond the current capabilities of standard optical microscopy setups. Consequently, the observed transient quenching events are interpreted as indirect evidence for the existence of the MacMillan conformer during discrete observational windows. Notably, the developed approach does not enable the direct capture of individual conformational switching events, only the detection of their averaged photophysical consequences over longer observational intervals.

**Comparison with Previous Reports and Interpretation.** To quantitatively assess the current observations, the relative populations of the **IM_2,Houk_** and **IM_2,Mac_** conformers were investigated under the present experimental conditions. The ratio between these conformers was determined to be ~6.6:1, indicating clear dominance by the Houk conformer (Figure S14E). This measured ratio aligns well with previously reported values derived from ensemble experimental studies; for instance, solution-phase NMR experiments have reported conformer ratios of ~3:1, while crystallographic studies have inferred ratios of up to 25:1.^[22,58]^ The current single-molecule measurement, which falls between these reported values, appears to be chemically reasonable, and supports the validity of the proposed photophysical interpretation.

However, potential uncertainties arising from experimental limitations must be acknowledged. More specifically, factors such as the molecular immobilisation on glass surfaces, potential surface-induced effects, and the limited temporal resolution may influence the observed conformer ratio. Thus, the obtained results, while supportive and consistent with chemical intuition and prior reports, should be considered as providing indirect, rather than definitive, evidence of conformational dynamics.

**Conclusion and Future Directions.** Our experimental results strongly suggest that single-molecule conformational changes can be indirectly supported by optical fluorescence microscopy through distinct fluorescence quenching events that are associated with particular conformational states (**IM_2,Mac_**). However, the current technological limitations regarding the temporal resolution prevent the direct, unequivocal observation of individual conformational transitions at the single-molecule level.

Therefore, the fundamental aim of directly observing single-molecule conformational changes via optical microscopy remains only partially unresolved. Addressing this challenge will require continued technological progress, such as the development of higher-temporal-resolution detectors capable of microsecond-scale single-molecule fluorescence imaging, or the employment of polarisation-sensitive microscopy methods. Further strategic experimental modifications, such as performing experiments under cryogenic conditions or designing structural analogues with higher rotational barriers, could also provide more direct conformational observations.

Additionally, the integration of single-molecule fluorescence studies with complementary ensemble techniques, such as time-resolved IR spectroscopy and advanced two-dimensional NMR techniques, could offer invaluable insights into conformational dynamics.^[22,58]^ The combined application of these diverse methodologies will be expected to significantly enhance our mechanistic understanding of single-molecule conformational processes and pave the way toward directly visualising structural changes in real time. Ultimately, continued advances along these directions hold substantial promise for revealing previously inaccessible details of conformational dynamics and reaction mechanisms at the single-molecule level, potentially catalysing significant breakthroughs in the fields of mechanistic organic chemistry and catalysis research.

**4. NMR Spectra**

^1^H-NMR spectra of **1** (CDCl_3_, 400MHz)

^1^H-NMR spectra of **2** (CDCl_3_, 400MHz)

^1^H-NMR spectra of **3** (CDCl_3_, 400MHz)

^1^H-NMR spectra of **4** (CDCl_3_, 400MHz)

^1^H-NMR spectra of **5** (CDCl_3_, 400MHz)

^1^H-NMR spectra of **6** (CDCl_3_, 400MHz)

cat_ctrl_

^1^H-NMR spectra of cat_ctrl_ (CDCl_3_, 400MHz)

**5. Computational Details**

All calculations were performed using the density functional theory^[64]^ (DFT) as implemented in the Gaussian 16^[65]^ suite of programs. Geometry optimizations were performed using the uB3LYP functional^[66,67]^ with Grimme’s D3 correction^[68]^ and def2-SVP^[69]^ basis set for all atoms. Vibrational frequency calculations were carried out at the same level of theory as that used for geometry optimizations, wherein thermochemistry correction energy (G – E) was acquired. Transition states were realized by the presence of single imaginary frequency and confirmed by intrinsic reaction coordinate calculations (IRC). Single point energies of optimized structures were calculated with the uM062X functional ^[66,67]^ with Grimme’s D3 correction^[68]^ and def2-TZVPP^[69]^ for all atoms. Solvation effects were incorporated using the SMD model^[70]^ based on the gas-phase optimized geometries and carried out at the same level as single-point calculations. Implicit ethanol solvent was modelled with the SMD continuum solvation^[71]^. Final solution phase Gibbs free energies were calculated as follows:

$G_{sol}=E_{sol}+\left( G-E \right)$, $\Delta G_{sol}$ for products – $\Delta G_{sol}$ for reactants

Where $G_{sol}$ is the final solution phase Gibbs free energy, $E_{sol}$ is the final solution phase total electronic energy and (G–E) is the thermal correction energy to Gibbs free energy.

**Noncovalent Interaction Analysis**

Noncovalent interactions were visualized by Multiwfn^[71]^ with .wfn file generated from the Gaussian 16 quantum chemical package at the uM062X-D3/ def2-TZVPP/SMD(EtOH)//uB3LYP-D3/def2-SVP level of theory. The NCI index is based on the correlation between the reduced density gradient and the electron density. The reduced density gradient (RDG) serves as tool to identify regions with noncovalent interactions in the system.

**Summarized Energy Components of All DFT-Optimized Structures**

**Table S1.** Summarized energy components of DFT-optimized structures

| **DFT-optimized**  **Structures** | **E(sol)** (SCF/TZ) [eV] uB3LYP-D3/def2-TZVPP, SMD(EtOH) | **G–E** (Thermochemistry correction energy) [eV] uB3LYP-D3/def2-SVP | **G(sol)** [eV] |
| --- | --- | --- | --- |
| **S** | -26930.71208 | 6.236498 | -26924.475584 |
| **IM_1_** | -45743.977022 | 13.835770 | -45730.141252 |
| **IM_2,Houk_** | -43627.718512 | 13.365530 | -43614.352982 |
| **IM_2,Mac_** | -43627.5798364 | 13.430619 | -43614.149216 |
| **TS_rot_** | -43627.581883 | 13.431517 | -43614.150365 |
| **IM_2_-cat_ctrl_** | -30659.108362 | 9.301562 | -30649.80679 |
| **diene** | -5281.419719 | 1.780577 | -5279.639141 |
| **P** | -32223.940136 | 8.821608 | -32215.118528 |

**Cartesian Coordinates of Optimized Geometries**

==============================================

**S**

==============================================

Charge: 0, Spin: 1

Cartesian coordinates:

ATOM X Y Z

F -0.818674000 4.257922000 -2.516019000

F -2.250598000 3.344019000 -0.988090000

O 2.496671000 -4.147732000 -2.722156000

N -0.332335000 4.569986000 -0.165354000

N -0.064676000 2.345624000 -1.244678000

C 1.721378000 -3.232433000 -2.903066000

C 1.723818000 -1.976218000 -2.149691000

C 0.826877000 -0.994585000 -2.405337000

C -1.947399000 6.408430000 -0.581069000

C 1.977762000 5.592428000 2.613888000

C -0.016966000 6.338163000 1.188250000

C 0.951250000 5.408457000 1.540538000

C 0.750041000 4.273117000 0.672579000

C -0.793838000 5.792561000 0.132952000

C 1.422117000 3.052131000 0.572926000

C 1.006399000 2.094262000 -0.389868000

C 1.506747000 0.810451000 -0.688117000

C 0.725000000 0.283783000 -1.736708000

C -0.233621000 1.283808000 -2.038367000

C 2.584543000 2.730952000 1.471645000

B -0.928226000 3.644263000 -1.277471000

H 3.390783000 3.469491000 1.348093000

H 0.922507000 -3.297929000 -3.692520000

H 2.489623000 -1.881046000 -1.373392000

H 0.091746000 -1.171244000 -3.201385000

H -1.732327000 6.472067000 -1.659289000

H -2.837381000 5.767360000 -0.481977000

H -2.165503000 7.409321000 -0.186668000

H 1.857938000 6.575429000 3.091009000

H 1.894700000 4.825146000 3.400461000

H 3.004356000 5.534684000 2.217512000

H -0.166905000 7.319686000 1.634767000

H 2.342169000 0.315293000 -0.200083000

H -1.022493000 1.265082000 -2.789879000

H 3.002488000 1.739202000 1.267775000

H 2.281237000 2.759174000 2.529029000

==============================================

**IM_1_**

==============================================

Charge: 1, Spin: 1

Cartesian coordinates:

ATOM X Y Z

F -5.337490000 4.525625000 -2.741655000

F -6.929424000 3.617803000 -1.372574000

O -4.915994000 -4.474860000 2.462572000

O -3.569398000 -4.297402000 -3.182253000

N -5.038469000 -5.799651000 0.578636000

N -4.467733000 -4.269031000 -0.996839000

N -4.969408000 4.604537000 -0.349015000

N -4.836627000 2.451434000 -1.582989000

C -5.395374000 -1.184254000 2.105909000

C -5.303602000 0.079875000 2.699608000

C -5.890446000 1.194579000 2.091180000

C -6.581045000 1.038730000 0.885122000

C -6.664826000 -0.224355000 0.293225000

C -6.069571000 -1.350236000 0.883761000

C -6.156279000 -2.706861000 0.212968000

C -5.248532000 -7.057365000 1.257134000

C -3.964586000 -6.676321000 -1.459290000

C -6.351223000 -5.857069000 -1.523502000

C -4.961769000 -5.659113000 -0.885217000

C -4.900898000 -4.624306000 1.252924000

C -4.813623000 -3.519513000 0.209771000

C -4.418180000 -3.596603000 -2.288677000

C -3.964635000 -2.178286000 -2.073191000

C -4.666128000 -1.092899000 -2.435132000

C -6.448991000 6.577158000 -0.654364000

C -2.785834000 5.180163000 2.649801000

C -4.634289000 6.202397000 1.195594000

C -3.743728000 5.178278000 1.499057000

C -3.956783000 4.156734000 0.507626000

C -5.375686000 5.820170000 0.050549000

C -3.370073000 2.898167000 0.325985000

C -3.826285000 2.053712000 -0.713358000

C -3.534231000 0.699034000 -0.980086000

C -4.402683000 0.269335000 -1.997423000

C -5.179821000 1.401298000 -2.340340000

C -2.310129000 2.391735000 1.262973000

B -5.562445000 3.825855000 -1.567112000

H -1.513483000 3.133633000 1.407504000

H -4.958510000 -2.057323000 2.598613000

H -4.775364000 0.189561000 3.651186000

H -5.810395000 2.183608000 2.550537000

H -7.029934000 1.901087000 0.386858000

H -7.187685000 -0.331907000 -0.660846000

H -6.140690000 -7.576550000 0.870006000

H -4.381271000 -7.731207000 1.157715000

H -5.398293000 -6.834385000 2.322371000

H -4.304863000 -7.704707000 -1.263080000

H -3.870001000 -6.540868000 -2.542363000

H -2.978982000 -6.532346000 -0.991830000

H -6.295033000 -5.728286000 -2.614744000

H -6.731594000 -6.872019000 -1.331631000

H -7.071465000 -5.132472000 -1.116420000

H -2.708742000 -4.379037000 -2.744133000

H -5.396839000 -3.589747000 -2.808857000

H -3.031730000 -2.069410000 -1.508321000

H -5.587095000 -1.235264000 -3.013849000

H -6.167789000 6.741876000 -1.706443000

H -7.378633000 5.987289000 -0.672149000

H -6.632603000 7.543433000 -0.166582000

H -2.911410000 6.093906000 3.248090000

H -2.939069000 4.316720000 3.316510000

H -1.736330000 5.143378000 2.313882000

H -4.755771000 7.137981000 1.739033000

H -2.838200000 0.077176000 -0.424240000

H -5.984565000 1.478513000 -3.070734000

H -1.849428000 1.467508000 0.897450000

H -2.751177000 2.178403000 2.250269000

H -6.495819000 -2.572673000 -0.822589000

H -6.936166000 -3.308472000 0.712057000

H -3.995732000 -2.839009000 0.486228000

==============================================

**IM_2,Houk_**

==============================================

Charge: 1, Spin: 1

Cartesian coordinates:

ATOM X Y Z

F 7.196824000 4.307391000 0.947137000

F 5.704127000 4.335469000 2.678863000

O 2.429057000 -6.468344000 -1.427680000

N 5.344376000 2.763729000 0.886723000

N 4.987229000 5.201663000 0.535386000

N 4.592506000 -4.354597000 0.356958000

N 4.073860000 -6.572294000 0.187525000

C 2.518818000 3.265719000 -1.649460000

C 5.817975000 1.611387000 1.338007000

C 5.115366000 0.529006000 0.732064000

C 4.156995000 1.133243000 -0.140899000

C 4.314575000 2.511078000 -0.029936000

C 3.614689000 3.592772000 -0.672803000

C 5.107328000 6.528351000 0.630291000

C 3.960907000 4.898850000 -0.377917000

C 3.427651000 6.165692000 -0.864222000

C 4.149654000 7.152091000 -0.228250000

C 2.325458000 6.388246000 -1.847558000

C 6.107659000 7.183927000 1.512717000

C 5.377731000 -0.832471000 0.996602000

C 4.732249000 -1.925581000 0.440447000

C 5.103707000 -3.228607000 0.809165000

C 3.561151000 -4.456140000 -0.680533000

C 3.245116000 -5.950612000 -0.698182000

C 4.994805000 -5.697291000 0.907759000

C 6.459748000 -6.007437000 0.575564000

C 4.731037000 -5.744300000 2.419563000

C 4.099243000 -8.016911000 0.323401000

C 3.989489000 -3.990541000 -2.095960000

C 5.309472000 -4.577145000 -2.536796000

C 6.500018000 -3.851762000 -2.374458000

C 7.733903000 -4.418630000 -2.708020000

C 7.791551000 -5.723228000 -3.208027000

C 6.609420000 -6.451532000 -3.383484000

C 5.376410000 -5.882638000 -3.052665000

B 5.874457000 4.180238000 1.318927000

H 6.181261000 -1.030177000 1.716691000

H 3.928675000 -1.771011000 -0.280067000

H 5.891469000 -3.339315000 1.561305000

H 1.558907000 3.689718000 -1.318993000

H 2.735962000 3.690831000 -2.640448000

H 2.381678000 2.186048000 -1.772741000

H 6.628907000 1.577326000 2.065717000

H 3.438849000 0.615613000 -0.770818000

H 4.026755000 8.227168000 -0.345936000

H 1.376342000 5.946050000 -1.504804000

H 2.555000000 5.939355000 -2.827223000

H 2.161058000 7.463649000 -1.999636000

H 6.041808000 8.276572000 1.443716000

H 7.121730000 6.852513000 1.237422000

H 5.952670000 6.864527000 2.555587000

H 6.626219000 -6.003166000 -0.508988000

H 6.730312000 -6.994298000 0.977162000

H 7.128894000 -5.270020000 1.042617000

H 3.684734000 -5.485833000 2.637801000

H 5.395179000 -5.057360000 2.964091000

H 4.930797000 -6.755380000 2.801196000

H 3.286905000 -8.416267000 -0.297633000

H 3.934886000 -8.325908000 1.366918000

H 5.054779000 -8.434135000 -0.031970000

H 6.460710000 -2.829980000 -1.985567000

H 8.651892000 -3.839216000 -2.582391000

H 8.754104000 -6.167645000 -3.471992000

H 6.646927000 -7.465597000 -3.788881000

H 4.454609000 -6.452557000 -3.193354000

H 3.170122000 -4.312015000 -2.757432000

H 4.027163000 -2.892900000 -2.134151000

H 2.659244000 -3.904354000 -0.365489000

==============================================

**IM_2,Mac_**

==============================================

Charge: 1, Spin: 1

Cartesian coordinates:

ATOM X Y Z

F 7.111188000 4.229538000 1.707779000

F 5.255803000 4.264277000 3.043878000

O 2.385172000 -6.460756000 -1.179001000

N 5.329918000 2.672667000 1.234658000

N 5.055584000 5.105233000 0.781842000

N 4.709913000 -4.438677000 0.498691000

N 3.997378000 -6.607862000 0.467436000

C 3.270357000 3.127024000 -1.961047000

C 5.673196000 1.524933000 1.802180000

C 5.146704000 0.436358000 1.046116000

C 4.443329000 1.029279000 -0.047153000

C 4.570135000 2.407610000 0.085838000

C 4.062058000 3.476464000 -0.730594000

C 5.130458000 6.433732000 0.898558000

C 4.305449000 4.788441000 -0.365388000

C 3.898915000 6.046330000 -0.977447000

C 4.417810000 7.043518000 -0.179720000

C 3.080541000 6.251902000 -2.210323000

C 5.864652000 7.103744000 2.003889000

C 5.336903000 -0.927130000 1.355486000

C 4.822294000 -2.003132000 0.648485000

C 5.124677000 -3.310654000 1.048920000

C 3.791789000 -4.553906000 -0.641531000

C 3.262440000 -5.981803000 -0.498662000

C 5.032155000 -5.784903000 1.089486000

C 6.445674000 -6.212700000 0.665288000

C 4.883409000 -5.781903000 2.615252000

C 3.806288000 -8.011625000 0.785198000

C 4.413880000 -4.399301000 -2.051675000

C 4.745736000 -2.989899000 -2.476837000

C 3.726133000 -2.133874000 -2.926289000

C 4.013942000 -0.818327000 -3.302034000

C 5.326597000 -0.338420000 -3.228322000

C 6.348676000 -1.182705000 -2.784262000

C 6.058573000 -2.499996000 -2.416039000

B 5.738762000 4.095764000 1.762760000

H 5.958850000 -1.138703000 2.233961000

H 4.205545000 -1.829114000 -0.232559000

H 5.767339000 -3.433286000 1.925734000

H 2.235728000 3.493882000 -1.879761000

H 3.708203000 3.592101000 -2.855979000

H 3.231109000 2.045530000 -2.131315000

H 6.271371000 1.499644000 2.713146000

H 3.931672000 0.498378000 -0.843565000

H 4.312596000 8.116699000 -0.328013000

H 2.088269000 5.780898000 -2.125796000

H 3.567240000 5.821315000 -3.100317000

H 2.930780000 7.324385000 -2.394876000

H 5.801148000 8.195331000 1.917533000

H 6.920892000 6.789910000 1.993459000

H 5.457540000 6.779902000 2.974743000

H 6.531280000 -6.244755000 -0.429962000

H 6.679691000 -7.210523000 1.063327000

H 7.193779000 -5.509410000 1.060996000

H 3.892422000 -5.406577000 2.908766000

H 5.663227000 -5.179787000 3.102435000

H 4.997323000 -6.807242000 2.993403000

H 3.063952000 -8.406936000 0.079374000

H 3.425955000 -8.148833000 1.809532000

H 4.744128000 -8.577268000 0.670314000

H 2.700345000 -2.507192000 -3.000477000

H 3.212713000 -0.172627000 -3.671445000

H 5.554793000 0.688109000 -3.524923000

H 7.376972000 -0.817171000 -2.733129000

H 6.865040000 -3.157636000 -2.079539000

H 5.303399000 -5.044495000 -2.115744000

H 3.660642000 -4.827588000 -2.731797000

H 2.957986000 -3.844631000 -0.520151000

==============================================

**TS_rot_**

==============================================

Charge: 1, Spin: 1

Imaginary frequency: –55.61 cm^–1^

Cartesian coordinates:

ATOM X Y Z

F 8.403595000 3.497664000 0.736500000

F 7.367681000 3.733649000 2.757091000

O 0.820356000 -5.490529000 -1.236872000

N 6.264347000 2.419464000 1.054856000

N 6.419971000 4.890432000 0.847839000

N 3.773214000 -4.220827000 0.188257000

N 2.708093000 -6.215650000 -0.134355000

C 3.094356000 3.728683000 -0.636451000

C 6.553179000 1.145715000 1.327535000

C 5.488465000 0.309647000 0.902143000

C 4.511137000 1.176488000 0.337165000

C 5.006719000 2.477815000 0.439317000

C 4.440498000 3.724636000 0.023732000

C 6.875804000 6.151917000 0.916824000

C 5.153304000 4.899522000 0.234507000

C 4.835981000 6.275512000 -0.083140000

C 5.916142000 7.029307000 0.347715000

C 3.608847000 6.813735000 -0.739831000

C 8.193024000 6.506409000 1.509943000

C 5.478431000 -1.109523000 1.010832000

C 4.456636000 -1.930237000 0.593047000

C 4.610755000 -3.328056000 0.665999000

C 2.528775000 -3.914949000 -0.526632000

C 1.888296000 -5.287635000 -0.674778000

C 3.903649000 -5.682299000 0.521867000

C 5.187475000 -6.282896000 -0.050831000

C 3.822659000 -5.869159000 2.042363000

C 2.409474000 -7.635162000 -0.151019000

C 2.625375000 -3.203270000 -1.928149000

C 3.984012000 -2.737002000 -2.394369000

C 4.274923000 -1.368027000 -2.512236000

C 5.540069000 -0.934120000 -2.918052000

C 6.539512000 -1.867572000 -3.212886000

C 6.255735000 -3.235470000 -3.121939000

C 4.985709000 -3.663660000 -2.724869000

B 7.169999000 3.639800000 1.376639000

H 6.376705000 -1.571127000 1.436183000

H 3.545617000 -1.504771000 0.172991000

H 5.501134000 -3.722145000 1.165645000

H 2.389755000 4.365430000 -0.080633000

H 3.165707000 4.140231000 -1.655218000

H 2.663244000 2.723942000 -0.705783000

H 7.488502000 0.858033000 1.808175000

H 3.560841000 0.879802000 -0.099285000

H 6.027177000 8.109789000 0.270992000

H 2.701438000 6.576657000 -0.160910000

H 3.464865000 6.386198000 -1.745257000

H 3.678212000 7.906534000 -0.837782000

H 8.353355000 7.591872000 1.478370000

H 9.007470000 6.003479000 0.964217000

H 8.249609000 6.160775000 2.554555000

H 5.199173000 -6.220420000 -1.146432000

H 5.265193000 -7.338287000 0.247000000

H 6.067394000 -5.759580000 0.349752000

H 2.908546000 -5.401614000 2.437949000

H 4.699156000 -5.424366000 2.535024000

H 3.810566000 -6.941166000 2.285358000

H 1.449723000 -7.771738000 -0.665271000

H 2.325817000 -8.037336000 0.870291000

H 3.186941000 -8.193970000 -0.694024000

H 3.503897000 -0.632400000 -2.267598000

H 5.747337000 0.136729000 -2.994510000

H 7.533045000 -1.531429000 -3.521806000

H 7.025365000 -3.972694000 -3.366710000

H 4.763940000 -4.731840000 -2.673294000

H 2.218430000 -3.910149000 -2.666756000

H 1.935226000 -2.349753000 -1.902829000

H 1.888835000 -3.307488000 0.132624000

==============================================

**IM_2_-cat_ctrl_**

==============================================

Charge: 1, Spin: 1

Cartesian coordinates:

ATOM X Y Z

F 5.753276000 4.327708000 2.411329000

F 7.269634000 4.270034000 0.700990000

N 4.565675000 -4.360315000 0.137852000

N 5.077163000 5.191907000 0.255118000

N 5.398396000 2.751098000 0.621288000

C 4.988509000 -5.709538000 0.578500000

C 3.297062000 -5.936920000 -1.101991000

C 3.444193000 -4.435771000 -0.822143000

C 5.108925000 -3.241977000 0.570076000

C 4.736916000 -1.937986000 0.199457000

C 5.397311000 -0.845059000 0.737788000

C 6.240118000 7.162725000 1.205597000

C 2.420128000 6.403463000 -2.120309000

C 4.271262000 7.149603000 -0.523428000

C 3.524717000 6.170319000 -1.142004000

C 4.037251000 4.899023000 -0.645701000

C 5.223130000 6.516987000 0.334980000

C 3.665063000 3.596355000 -0.924520000

C 4.355503000 2.508342000 -0.282854000

C 4.181851000 1.131718000 -0.389799000

C 5.144370000 0.518456000 0.471528000

C 5.865881000 1.594014000 1.067117000

C 2.552808000 3.280320000 -1.886257000

C 3.801298000 -6.593038000 0.191902000

B 5.940691000 4.162372000 1.054764000

H 6.207382000 -1.045009000 1.449951000

H 3.922547000 -1.786530000 -0.511839000

H 5.926338000 -3.366797000 1.290171000

H 5.217987000 -5.704552000 1.653730000

H 5.900696000 -5.997064000 0.027609000

H 3.026942000 -6.549946000 0.975110000

H 4.090997000 -7.644770000 0.062392000

H 2.263695000 -6.209993000 -1.355606000

H 3.936856000 -6.226540000 -1.951023000

H 3.672003000 -3.841400000 -1.720040000

H 2.543836000 -4.008880000 -0.348001000

H 6.079151000 6.862472000 2.253398000

H 7.245941000 6.804336000 0.934511000

H 6.198286000 8.255477000 1.121126000

H 2.279110000 7.480001000 -2.287216000

H 2.631382000 5.934889000 -3.094817000

H 1.463790000 5.988594000 -1.763592000

H 4.168993000 8.225456000 -0.653180000

H 3.452249000 0.620709000 -1.011893000

H 6.685182000 1.551853000 1.784972000

H 2.384847000 2.202569000 -1.986083000

H 2.774184000 3.679188000 -2.887375000

H 1.607728000 3.737479000 -1.557849000

==============================================

**diene**

==============================================

Charge: 0, Spin: 1

Cartesian coordinates:

ATOM X Y Z

C -2.484170000 0.045863000 -0.004175000

C -3.080412000 -1.168391000 0.000597000

C -4.539915000 -0.991835000 0.001261000

C -4.829418000 0.329573000 -0.003110000

C -3.544837000 1.113223000 -0.006982000

H -1.412191000 0.247872000 -0.005823000

H -2.572994000 -2.135182000 0.003535000

H -5.263231000 -1.809740000 0.004758000

H -5.822305000 0.781373000 -0.003820000

H -3.465176000 1.775006000 -0.891310000

H -3.463628000 1.781188000 0.872544000

==============================================

**P**

==============================================

Charge: 0, Spin: 1

Cartesian coordinates:

ATOM X Y Z

F 7.311227000 2.350764000 0.379174000

F 6.975930000 0.824244000 -1.287506000

O 5.469863000 -4.641626000 2.709078000

N 5.396245000 2.647219000 -1.072238000

N 5.463409000 0.807244000 0.598709000

C 4.316936000 -4.286570000 2.697395000

C 3.816585000 -2.916450000 3.100241000

C 4.920448000 -1.817358000 3.154646000

C 7.195845000 3.700995000 -2.421895000

C 2.130108000 4.301645000 -1.898059000

C 4.642329000 4.283211000 -2.415694000

C 3.530890000 3.798419000 -1.733427000

C 4.015830000 2.751508000 -0.874284000

C 5.777683000 3.553836000 -1.986455000

C 3.357285000 1.911746000 0.036554000

C 4.091507000 0.948147000 0.763528000

C 3.673986000 -0.011081000 1.724877000

C 4.806371000 -0.725722000 2.133581000

C 5.886434000 -0.179128000 1.402928000

C 1.872444000 2.022793000 0.251832000

C 3.296038000 -2.968475000 4.602613000

C 2.567727000 -1.654831000 4.835273000

C 4.850391000 -1.343212000 4.662929000

C 3.496159000 -0.684870000 4.874025000

C 4.616920000 -2.702298000 5.357520000

B 6.362888000 1.648712000 -0.353694000

H 3.502478000 -5.007886000 2.418038000

H 2.982645000 -2.656453000 2.429954000

H 7.835883000 3.925923000 -1.554365000

H 7.564252000 2.750316000 -2.838059000

H 7.295946000 4.496800000 -3.171680000

H 2.102194000 5.113547000 -2.638906000

H 1.443261000 3.511478000 -2.242387000

H 1.717488000 4.695535000 -0.955046000

H 4.651480000 5.083664000 -3.153732000

H 2.658773000 -0.161122000 2.081833000

H 6.937547000 -0.465343000 1.430407000

H 1.508789000 1.300565000 0.991109000

H 1.326074000 1.850950000 -0.687856000

H 1.601231000 3.030728000 0.600630000

H 5.892593000 -2.313930000 3.017894000

H 3.325058000 0.390719000 4.935299000

H 1.482065000 -1.539655000 4.854333000

H 5.728237000 -0.758792000 4.967903000

H 5.402911000 -3.444107000 5.147954000

H 4.468533000 -2.611753000 6.443644000

H 2.740038000 -3.883154000 4.852759000

**6. Supporting Movie Legend**

**Movie S1.** Real–time observation of iminium ion formation at single–molecule level. (A) Catalytic reactions were monitored at the single-molecule level using TIRF microscopy, with the functionalized optical probe immobilized on a glass surface. (B) Time–dependent changes in fluorescence intensity over time allowed for tracking the reaction process. Individual signals were analyzed using hidden Markov model (HMM) analysis, providing detailed insights into molecular kinetics supported by quantum chemical calculations. (C) HMM-estimated states were assigned to putative intermediates in iminium catalysis. This analysis provided deeper insights into key aspects of the reaction, such as reversibility, intermediate states, and the transition probabilities and rates between different states.
